# RETRACTED: Antimicrobial effect of blue light on antibiotic-sensitive and drug-resistant Escherichia coli: a novel isotropic optical fibre

**DOI:** 10.1099/acmi.0.000967.v3

**Published:** 2025-03-19

**Authors:** Megan H. Goh, Joseph J. Connolly, Antonia F. Chen, Robert A. Rabiner, Santiago A. Lozano-Calderon

**Affiliations:** 1 Division of Orthopaedic Oncology, Department of Orthopaedic Surgery, Massachusetts General Hospital, Harvard Medical School, Boston, MA, USA; 2 Department of Orthopaedic Surgery, University of Texas Southwestern Medical Center, Dallas, TX, USA; 3 ABL Medical Inc., East Providence, RI, USA

**Keywords:** antimicrobial blue light, *Escherichia coli*, optical fiber, pelvic reconstruction

## Abstract

This article has been retracted. The retraction notice can be found at 10.1099/acmi.0.001223

**Background.** Orthopaedic oncological pelvic reconstructions have an elevated risk of infection with Gram-negative bacteria. This study evaluates the bactericidal ability of a novel antimicrobial blue light (ABL)-emitting optical fibre on antibiotic-sensitive *Escherichi coli* (AS-Ec) and ESBL-producing *E. coli* (ESBL-Ec).

**Methods.** Time-to-kill assays used a 10 ml NaCl solution with a starting inoculum of 1×10^5^ c.f.u. ml^−1^ for AS-Ec or ESBL-Ec; assays were repeated at least three times per strain. Experimental tubes had either one optical fibre [20.1 mW mm^−1^; low power (LP)] or two optical fibres [40.3 mW mm^−1^; high power (HP)], which delivered five wavelengths of ABL over 60 min. Control tubes had no optical fibres. Fifty microlitres of samples taken from each tube at 0, 10, 20, 30 and 60 min were streaked onto agar plates and incubated. c.f.u. ml^−1^ was determined. Bactericidal reduction was defined as a 99.9% (≥3 log_10_) reduction in c.f.u. ml^−1^. One-way ANOVA was conducted.

**Results.** Bactericidal effects were seen for AS-Ec under both LP-ABL and HP-ABL with a log_10_c.f.u. ml^−1^±sd difference of 3.44±0.35 (*P*=0.043) and 3.74±0.21 (*P*=0.048) at 30 and 20 min, respectively. For ESBL-Ec, while there was a significant reduction in bacterial colony formation, the bactericidal threshold was not reached with a log_10_c.f.u. ml^−1^±sd difference of only 1.02±0.41 (*P*=0.034) and 2.53±0.22 (*P*=0.037) at 60 min for LP-ABL and HP-ABL, respectively.

**Conclusions.** A novel ABL-emitting optical fibre exhibited bactericidal effects in AS-Ec and a clinically meaningful reduction of ESBL-Ec, providing a promising avenue for the use of ABL as a potential therapy for Gram-negative infections.

## Data Summary

All data associated with this work are reported within the article and supplementary materials.

## Introduction

Periprosthetic joint infections (PJIs) are a common and burdensome complication of endoprosthetic reconstruction following pelvic tumour resection with an infection rate of up to 47% [1]. Orthopaedic oncology patients are already at an elevated risk of infection due to impaired, dysregulated immune systems further compromised by immunosuppressive therapies. Furthermore, the complex nature of pelvic reconstruction often creates large dead spaces with limited soft tissue coverage exacerbating infection risk [2]. These dead spaces provide an alcove for bacterial growth and hinder the penetration and efficacy of systemic antibiotics. Moreover, the pelvis is closely associated with the gastrointestinal and genitourinary systems, which explains the unusually high proportion of polymicrobial and Gram-negative infections in pelvic PJI [2]. Importantly, PJIs caused by Gram-negative organisms have been traditionally considered to be more challenging to manage and result in less optimal outcomes [3].

*Escherichia coli* is one of the most common Gram-negative bacteria in the gastrointestinal tract and is often implicated in orthopaedic implant infections [4]. Concerningly, Cremet *et al*. [5] examined 30 clinical *E. coli* isolates that were recovered from deep periprosthetic infections and found that 56.7% of the *E. coli* strains had a high number of virulence-associated genes. These genes help a micro-organism invade the host, cause pathologic damage and evade host defences [6]. Moreover, the growing incidence of *E. coli* that can produce extended-spectrum beta-lactamases (ESBL) further complicates the treatment of Gram-negative PJIs in orthopaedic oncology patients [7]. ESBLs are enzymes that enable resistance against all cephalosporins, penicillins and aztreonam, as well as frequently having cross-resistance against quinolones and trimethoprim/sulfamethoxazole [8].

Although prophylactic antibiotics are a cornerstone of orthopaedic oncology, there are two major issues with this practice: (1) the lack of consensus on the optimal regimen as drug combinations differ based on institution [9] and (2) the growing crisis of antimicrobial resistance (AMR), which has been projected to become one of the leading primary causes of deaths globally by 2050 [10,11]. Once an orthopaedic oncology patient gets a postoperative infection, treatment can often include a combination of invasive surgical procedures and powerful systemic antibiotics [1]. While there have been numerous innovations to prevent and treat orthopaedic infections, many of these approaches still rely on the use of antibiotics [12,15]. In accordance with good antibiotic stewardship, it is imperative to explore alternative antimicrobial therapies.

Antimicrobial blue light (ABL), which is part of the visible light spectrum and includes the wavelengths between 400 and 470 nm, is a novel potential treatment approach [16]. ABL causes microbes’ death through the photoexcitation of endogenous microbial porphyrins and the generation of reactive oxygen species (ROS), which cause DNA damage, lipid peroxidation and membrane damage without causing harm to host tissue [17]. Preclinical studies have shown promise in the treatment of urogenital [18,19] and intestinal infections [20], which offers an encouraging potential avenue to treat Gram-negative orthopaedic infections such as *E. coli*. However, there are logistical challenges to the administration of ABL for indwelling infections, as the implementation of ABL has been limited to easily accessible superficial infections in the mouth [21,23] and on the skin [24,25].

In this study, we evaluated a patented, novel isotropic optical fibre that radially emits ABL with an equal and even power distribution along the entire length of the fibre, which could enable ABL exposure to luminal anatomic locations such as the dead spaces created after pelvic reconstruction. This study aims to be a preliminary, proof-of-concept study in which the bactericidal effect of this novel isotropic optical fibre is evaluated on antibiotic-sensitive *E. coli* (AS-Ec) and ESBL-producing *E. coli* (ESBL-Ec) under clinically relevant *in vitro* conditions.

## Methods

### Optical fibre design

Prior preclinical research conducted to investigate the efficacy of ABL used single point illumination [21,23] or variations of single point illumination [22,24, 25], which is a reasonable approach for localized, superficial dental and cutaneous infections. However, to treat luminal infections in locations such as pelvic reconstruction cavities, single point illumination would be impractical due to the inverse square law, which states that the intensity of a light source reduces at a rate proportional to the square of the distance between the source and receiver [26]. As a result, an attempt to directly illuminate a cavity with only single point illumination results in an unequal distribution of light exposure, with the highest intensity exposure occurring closest to the source and a substantially reduced amount of exposure to the more distant regions of the desired treatment area. To overcome the limitations of single point illumination, a novel optical fibre was designed to enable equal light intensity and power distribution over the entirety of the active length of the fibre.

The polymer optical fibre (POF) had two components: (1) a transparent polymethyl methacrylate (PMMA) core capable of transmitting visible light with minimal optical losses and (2) a thin cladding made up of a fluorinated polymer with a thickness of 20 µm that surrounded the PMMA core. The cladding had a lower refractive index than the PMMA core, which facilitated the transmission of light through the optical core by total internal reflection by Snell’s law.

**Fig. 1.**
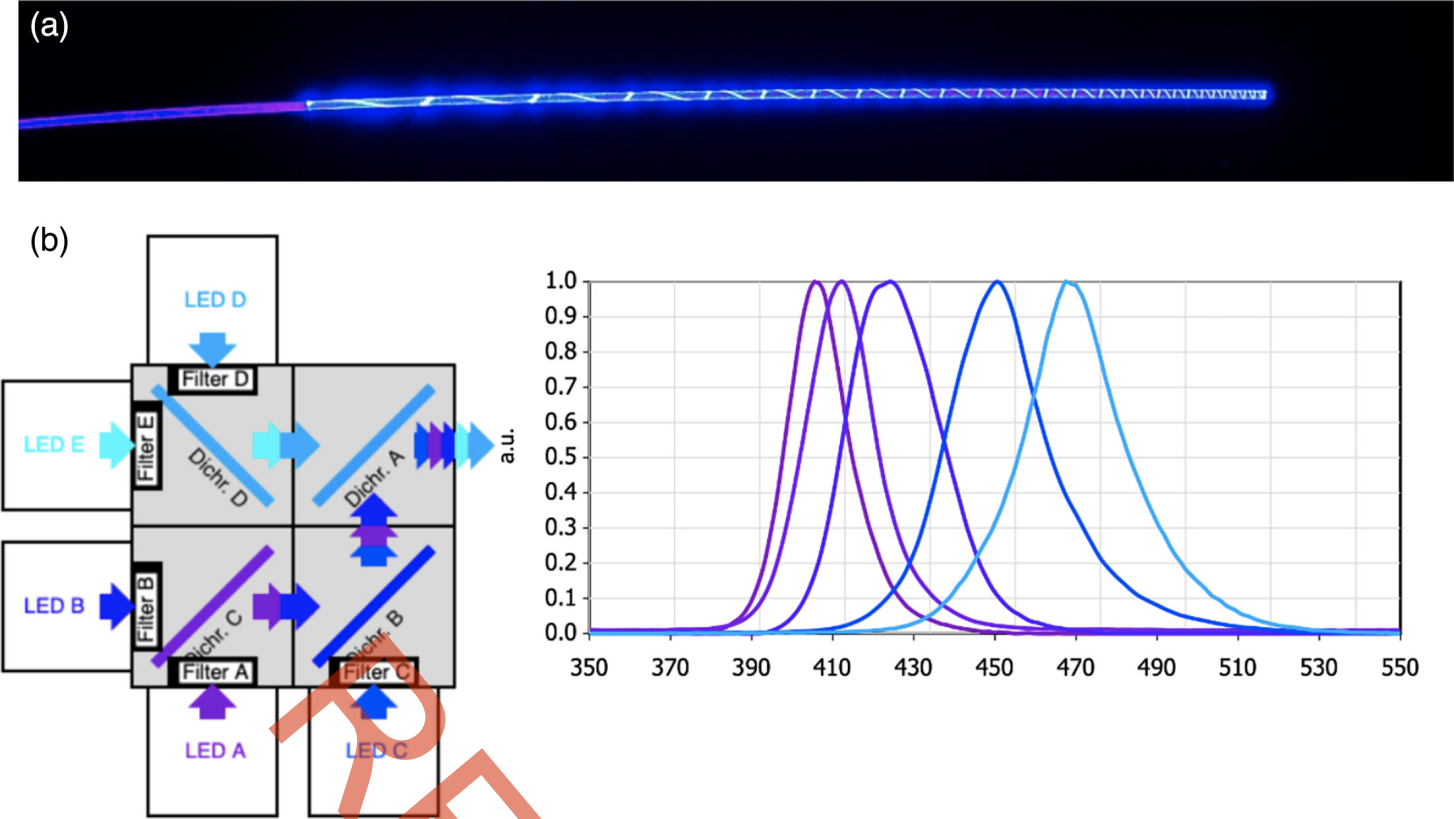
(a) A visual demonstration of the optical fibre illuminated. (b) Multi-wavelength blue light source design and output.

Equal and even ABL distribution along the length of the active emissive fibre was achieved by incrementally removing a thin ribbon of cladding in a circumferential helical manner in an increasingly tighter spiral fashion along the length of the POF, allowing for both linear and 360° radial emittance of ABL without aggregative loss of power over distance (Fig. 1).

### Blue light source

To facilitate the delivery of multiple ABL light frequencies (405–475 nm) through the same fibre, a novel light motor was designed to enable control over the optical output power of the light-emitting diodes (LEDs). The light from five modular high-powered LEDs (405, 415, 435, 450 and 475 nm) was reflected and combined with dichromic mirrors into a single non-coherent light beam that was transmitted through the POF. The mirrors were positioned to allow for the collimation of the beams in an additive fashion (Fig. 1b). The formation of a block power (square wave) was achieved through the modulation of the power applied to each of the LEDs. The output power of the system was measured with an optical power metre (Thorlabs PM100D) with a photodiode power sensor (Thorlabs S142C). The optical frequencies of the outputs were confirmed with a spectrometer (Ocean Optics Flame and Oceanview software).

**Fig. 2.**
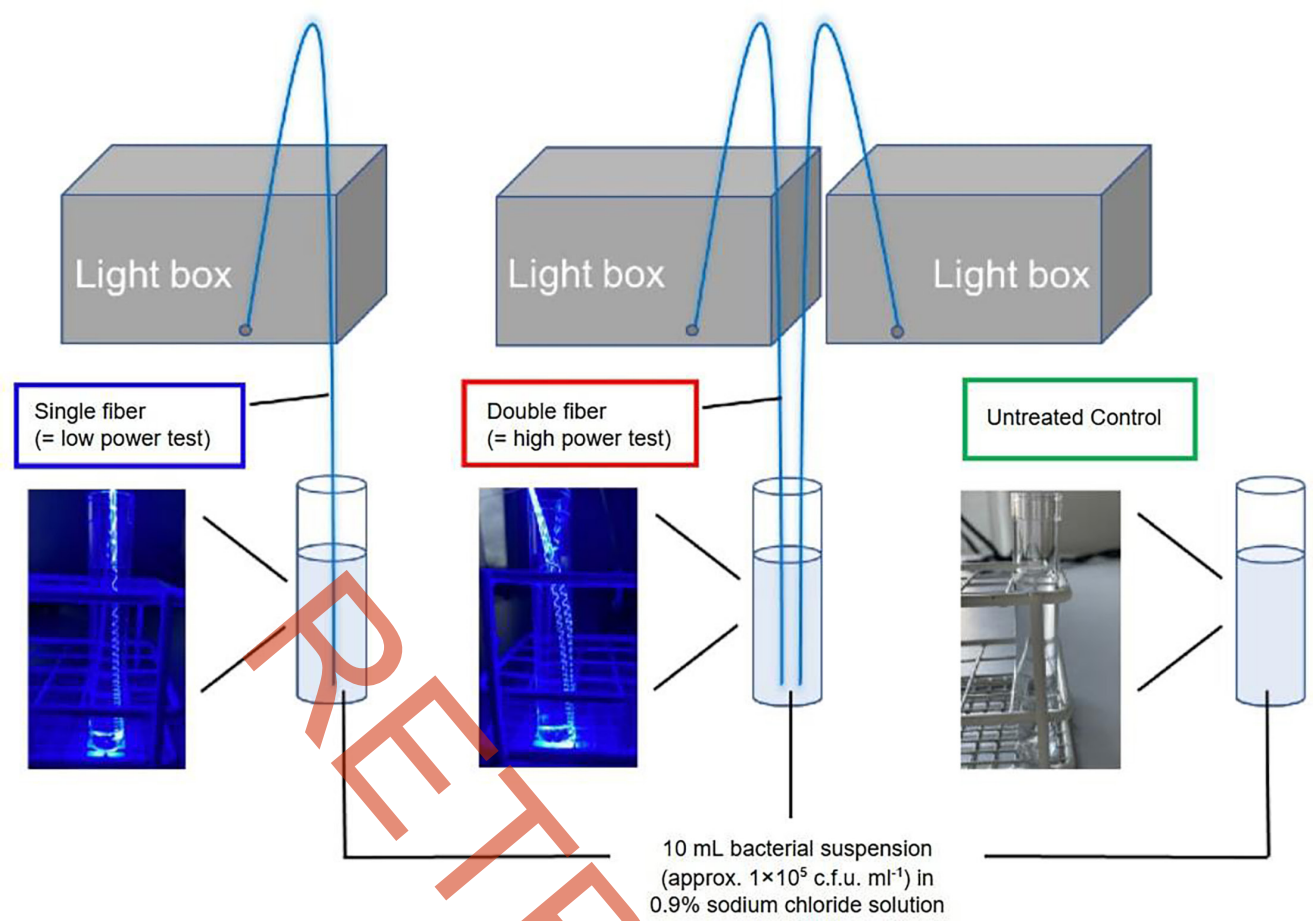
Experimental set-up for time-to-kill assays.

### Bacterial suspension preparation

Bacterial strains of *E. coli* ATCC 25922 (AS-Ec) and *E. coli* AR11 (ESBL, 3MRGN) (ESBL-Ec) were obtained from a commercial bacterial supplier (Leibniz Institute DSMZ). Both bacterial strains were prepared per Wisplinghoff protocol. Strains were diluted 1:10, plated and incubated for ~24 h in Mueller–Hinton broth prior to testing. A sample of each respective bacterium was taken from the fresh overnight cultures using a sterile inoculation loop, and each was mixed with 2 ml of API® NaCl 0.85% (bioMérieux Inc.) in their respective sterile container. The McFarland value (McF), a measurement of bacterial cell concentration, was obtained for each bacterial suspension from a densitometer [27]. Bacterial suspensions were adjusted through the addition of more NaCl or bacterial sample from the overnight cultures to achieve ~0.5 McF, which approximated a bacterial cell concentration of 1.5×10^8^ c.f.u. ml^−1^ [28,29]. Serial dilutions were then done to make 30 ml of the final bacterial concentration 1.0×10^5^ c.f.u. ml^−1^. The final 30 ml of each respective bacterial strain was split into three 10-ml test tubes. All testing was conducted in Köln, Germany, at Labor Dr. Wisplinghoff, an accredited medical laboratory.

### Time-to-kill assays

Based on the degree of ABL exposure, there were three groups: (1) low power (LP-ABL; one optical fibre, 20.1 mW mm^−1^), (2) high power (HP-ABL; two optical fibres, 40.3 mW mm^−1^) and (3) control group (no optical fibre) with no ABL exposure (Fig. 2). The novel optical fibre (1.5 mm diameter and 10 cm of active light emitting length) delivered the ABL. Optical fibres were held in place in their respective test tubes with flexible arm(s). All time-to-kill assays were conducted at ambient room temperature throughout testing.

Five simultaneous and continuous wavelengths of ABL (405, 415, 435, 450 and 475) were delivered by each optical fibre over 60 min. Fifty microlitres of samples were taken from each test tube at five different time points (0, 10, 20, 30 and 60 min) and streaked onto Mueller–Hinton agar plates. The plates were incubated at 36±1 °C for up to 30 h, and the c.f.u. per millilitre (c.f.u. ml^−1^) at each time point for each strain was then determined. A minimum of three time-to-kill assays was done for each strain. The bactericidal effect was defined as a reduction of 99.9% (≥3 log_10_) in c.f.u. ml^−1^ [30].

### Heat dissipation measurements

The degree of heat dissipation was evaluated based on the degree of ABL exposure for four groups: (1) low power (LP-ABL; one optical fibre, 20.1 mW mm^−1^), (2) high power (HP-ABL; two optical fibres, 40.3 mW mm^−1^), (3) extra high power (XHP-ABL, 3 optical fibres, 60.3 mW mm^−1^) and (4) control group (no optical fibre). Due to lumen occlusion, XHP-ABL was not used in time-to-kill assays. A 4-channel electronic digital recording thermometer was used (Omega HH378; accuracy ±0.1% of reading +0.7 °C) with K-type mini connector thermocouples that were put in each test tube with 10 ml of normal saline. The thermocouples were placed at the same relative position and depth in each test tube. The diameter of the thermocouples was 0.381 mm, and they had a response time of ~0.4 s. Temperature measurements were recorded once every hour throughout 6 h.

### Statistical analysis

The differences between the bacterial concentrations of the samples at different time points for each strain were evaluated with one-way ANOVA. Temperature differences between the test tubes in the heat dissipation experiments were also evaluated by one-way ANOVA. *P*-values<0.05 were considered statistically significant. All statistical analyses were performed using STATA.

## Results

**Fig. 3.**
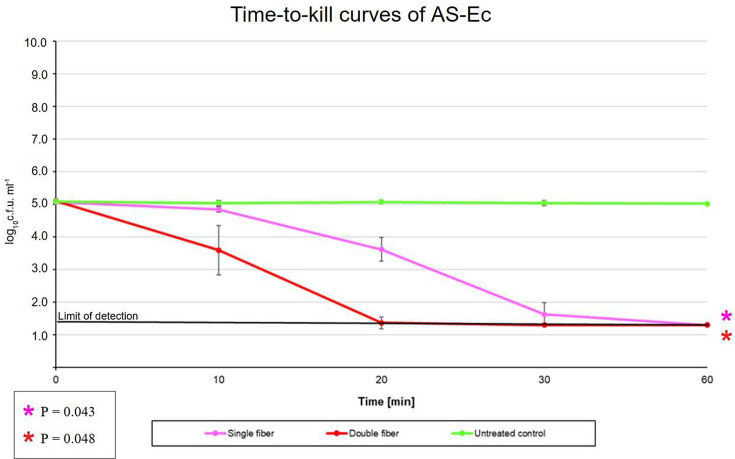
Time-to-kill curves for antibiotic-sensitive *E. coli*.

For AS-Ec, LP-ABL resulted in a log_10_c.f.u. ml^−1^±sd difference (percent reduction) of −0.23±0.08 (41.11 %), −1.45±0.36 (96.45 %), −3.44±0.35 (99.96%) and −3.77±0.02 (99.98 %) at 10, 20, 30 and 60 min, respectively (Fig. 3 and Table 1). For HP-ABL, there was a log_10_c.f.u. ml^−1^±sd difference (percent reduction) of −1.51±0.75 (96.91 %), −3.74±0.21 (99.98 %), −3.80±0.05 (99.99 %) and −3.80±0.05 (99.99%) at 10, 20, 30 and 60 min, respectively (Fig. 3 and Table 1). There was a statistically significant difference between the time points for both LP-ABL and HP-ABL exposed groups (*P*=0.043 and 0.048, respectively).

**Table 1.** AS-Ec concentration after ABL exposure

Time length of ABL exposure (min)	LP ABL (20 mW mm^−1^)		HP ABL (40 mW mm^−1^)	
	Log_10_ reduction	Percent reduction (%)	Log_10_ reduction	Percent reduction (%)
10	0.23	41.11	1.51	96.91
20	1.45	96.45	3.74	99.98
30	3.44	99.96	3.80	99.99
60	3.77	99.98	3.80	99.99

**Fig. 4.**
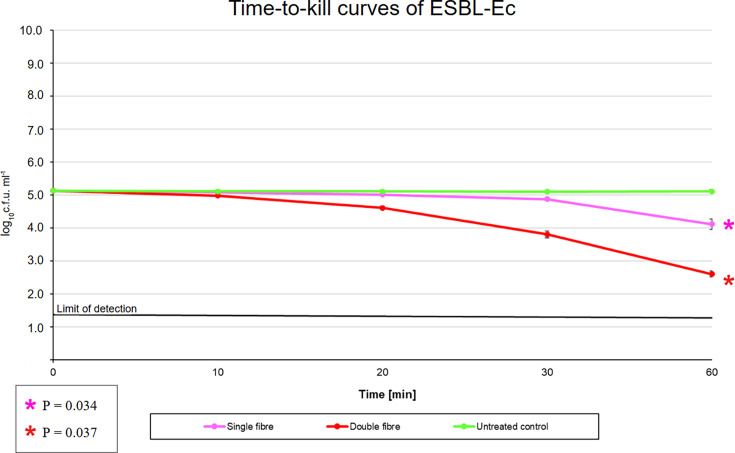
Time-to-kill curves for ESBL-Ec.

For ESBL-Ec, LP-ABL resulted in a log_10_c.f.u. ml^−1^±sd difference (percent reduction) of −0.04±0.02 (8.799 %), −0.12±0.06 (24.14 %), −0.25±0.15 (43.76 %) and −1.02±0.41 (90.45 %) at 10, 20, 30 and 60 min, respectively (Fig. 4 and Table 2). For HP-ABL, there was a log_10_c.f.u. ml^−1^±sd difference (percent reduction) of −0.15±0.04 (29.21 %), −0.52±0.09 (69.80 %), −1.33±0.12 (95.32 %) and −2.53±0.22 (99.70 %) at 10, 20, 30 and 60 min, respectively (Fig. 4 and Table 2). There was a statistically significant difference between the time points for both LP-ABL and HP-ABL exposed groups (*P*=0.034 and 0.037, respectively). Raw data for the AS-Ec and ESBL-Ec experiments can be found inFile S1 (available in the online Supplementary Material).

**Table 2.** ESBL-Ec concentration after ABL exposure

Time length of ABL exposure (min)	LP ABL (20 mW mm^−1^)		HP ABL (40 mW mm^−1^)	
	Log_10_ reduction	Percent reduction (%)	Log_10_ reduction	Percent reduction (%)
10	0.04	8.799	0.15	29.21
20	0.12	24.14	0.52	69.80
30	0.25	43.77	1.33	95.32
60	1.02	90.45	2.53	99.70

For the temperature studies, the average temperature±sd (range) was 22.2 °C±0.29 (0.9°C), 24.0 °C±1.16 (3.3°C), 24.4 °C±1.34 (4.0°C) and 24.8 °C±1.51 (4.3°C) for the control, LP-ABL, HP-ABL and XHP-ABL groups, respectively (*P*=0.002) (Table 3).

**Table 3.** Heat dissipation measurements

Time length of ABL exposure (hour)	Temp. (°C)			
	Control (no ABL)	LP ABL	HP ABL	Extra HP ABL
0	21.6	21.7	21.7	21.8
1	22.2	23.2	23.8	23.9
2	22.2	24.0	24.5	24.8
3	22.3	24.4	24.8	25.3
4	22.4	24.7	24.9	25.7
5	22.3	24.7	25.4	25.9
6	22.5	25.0	25.7	26.1

## Discussion

In this preliminary, proof-of-concept study, we evaluated the bactericidal ability of a novel isotropic optical fibre under clinically relevant *in vitro* conditions on AS-Ec and ESBL-Ec. A reduction in c.f.u. was seen at longer durations and higher intensities of ABL exposure. Longer exposures of LP-ABL and HP-ABL showed a significant reduction in bacterial formation. HP-ABL was able to reach the bactericidal threshold more quickly than LP-ABL in AS-Ec, as the bactericidal effect was seen at 20 and 30 min for HP-ABL and LP-ABL, respectively. Conversely, in ESBL-Ec, both LP-ABL and HP-ABL were unable to meet the bactericidal percentile reduction cutoff of 99.9%. However, HP-ABL nearly reached that threshold, achieving a percentile reduction of 99.7% at the 60 min time point. Regarding the heat dissipation studies, while a significant difference in the temperature between the groups was observed over the 6 h of measurement, the results must be appropriately contextualized for clinical relevance. Even after 6 h of exposure at the highest intensity of ABL with XHP-ABL, the average temperature was 24.8 °C±1.51, which remained far below the normal body temperature of 37°C [31]. Therefore, there is minimal to no concern for the optical fibre(s) causing tissue damage during treatment. Moreover, the temperatures across all the ABL-exposed groups were well below the 45–75 °C required to cause the thermal death of *E. coli* [32]. This absolves concerns that the heat generated by the optical fibre(s) could be a confounder for the antimicrobial effects observed in our study.

This study had several limitations. First, our study was conducted in an *in vitro* setting, which does not fully account for the multitude of factors that would be encountered in an *in vivo* setting; thus, further investigation is needed to explore if similar results can be achieved in experiments that better mimic the endogenous conditions of the body. Second, as this was a preliminary, proof-of-concept study rather than a comparator study, the antimicrobial performance of this novel optical fibre was not evaluated against other ABL delivery modalities, limiting the ability to assess its relative efficacy. Third, this study only evaluated the impact of ABL on one specific strain of AS-Ec and ESBL-Ec; thus, further evaluation is needed to investigate the potential therapeutic role of this novel ABL-emitting optical fibre on other strains of *E. coli* and ESBL-producing bacteria. Fourth, we only investigated the use of two different intensities of ABL (LP-ABL and HP-ABL) that were administered for a maximum of 60 min. Given that the reduction in bacterial formation was closely related to both light intensity and treatment duration, further investigation is needed to find the optimal combination to maximize the bactericidal capabilities of ABL.

The growing concern for AMR has spurred major research interest and exploration into alternative non-antibiotic therapeutic approaches such as ABL, which pose minimal risk for the development of bacterial resistance. The proposed mechanism of action is that ABL triggers the creation of cytotoxic ROS through the excitation of endogenous photosensitive microbial chromophores [17]. The ROS subsequently cause multitarget oxidative damage to numerous macromolecules such as proteins, nucleic acids and lipids, facilitating microbial damage and death [33]. Dos Anjos *et al*. [34] investigated the potential bacterial targets of ABL in * E. coli* and found that ABL exposure significantly increased ROS production with a significant degradation in bacterial proteins and DNA as well as lipid peroxidation. Further, there were ultrastructural changes to *E. coli*, including the detachment of membrane structures that caused elongation and irregularities in the cell membrane/wall. The extensive damage to *E. coli* following ABL exposure seen in Dos Anjos *et al*. [34] provides context and insight into our study, in which we achieved bactericidal effects after relatively short durations of ABL. Our findings support the potential use of ABL as a standalone treatment for this strain of AS-Ec.

There is a paucity of research investigating the potential efficacy of multiwavelength ABL as a standalone treatment for ESBL-Ec. Gardner *et al*. [35] examined the efficacy of both concurrent and individually administered far-ultraviolet C (UVC, 222 nm) and single wavelength ABL (405 nm) on two AS-Ec and two ESBL-Ec strains. Their study found a significant reduction of 5–6 log_10_c.f.u. ml^−1^ in all strains with dual exposure of UVC and single wavelength ABL. Interestingly, exposure to single wavelength ABL alone only resulted in a 0.18 log_10_c.f.u. ml^−1^ reduction in one of the AS-Ec strains, with no reduction seen in the other three strains. This discrepancy about our study is likely multifactorial: (1) different *E. coli* strains were evaluated, which could have inherently different ABL sensitivities, (2) multi-wavelength ABL was utilized in our study as opposed to a single wavelength of ABL and (3) the duration of ABL exposure in our study was double that used in Gardner *et al*. [35]. While other studies have seen comparable success to our study with the use of single wavelength ABL against both antibiotic-sensitive and multidrug-resistant *E. coli* [36,38], similar factors are likely driving the discrepancies in results, such as the exact *E. coli* strains that were tested, durations of ABL exposure and ABL intensity. In two separate studies that evaluated the antimicrobial effect of ABL on ESBL-Ec [39] and ESBL-producing *Klebsiella pneumoniae* [40], Dos Anjos *et al*. [39,40] found that ABL reduced the activity of *β*-lactamase. While the mechanism behind the reduction in *β*-lactamase activity has not been fully elucidated, it was postulated that ABL causes a conformational change in the enzyme, reducing its efficacy [39]. While we did not achieve bactericidal effects with either LP- or HP-ABL in ESBL-Ec, after 60 min of HP-ABL exposure, there was a 99.7% reduction in ESBL-Ec c.f.u., which is just below our 99.9% reduction cutoff and is still clinically meaningful. Further investigation is needed to determine if a longer duration of HP-ABL exposure could achieve bactericidal effects. Nevertheless, our study offers insight into the potential use of ABL as an augmentative therapy to significantly reduce the bacterial load of ESBL-Ec and can be used in tandem with other approaches for the eradication of the infection.

## Conclusion

ABL has shown promise as a therapeutic avenue for difficult-to-treat infections but has been limited in its application due to the logistical challenges of implementation. Our study has provided a clinically impactful means of overcoming this limitation with the development of a novel isotropic optical fibre that could reach indwelling infections that are otherwise resistant to systemic antibiotic therapy. This is of particular importance in orthopaedic oncology patients, who are at an exceptionally high risk of postoperative infection. In the case of pelvic endoprosthetic reconstructions, this novel optical fibre could address important concerns of polymicrobial, Gram-negative infections like *E. coli* and serve as a means to access deep dead space pockets. Future studies will focus on more accurately replicating *in vivo* cavitary conditions with additional consideration for the type of bacterial colony medium that is exposed to ABL and conducting comparator studies to evaluate the novel optical fibre against other ABL delivery modalities. While further exploration is needed to refine and optimize the use of ABL, this is an exciting next step to ABL becoming more widely used in a variety of clinical settings.

## Supplementary material

10.1099/acmi.0.000967.v3 Uncited Supplementary Material 1.

## Abbreviations

ABL: antimicrobial blue light
AMR: antimicrobial resistance
AS-Ec: antibiotic-sensitive *Escherichia coli*
ESBL: extended-spectrum beta-lactamases
ESBL-Ec: ESBL-producing *Escherichia coli*
HP: high power
LEDs: light-emitting diodes
LP: low power
McF: McFarland value
PJIs: periprosthetic joint infections
PMMA: polymethyl methacrylate
POF: polymer optical fibre
ROS: reactive oxygen species

## References

[1] Trovarelli G, Angelini A, Pala E, Cappellari A, Breda A (2019). Infection in orthopaedic oncology: crucial problem in modern reconstructive techniques. Eur Rev Med Pharmacol Sci.

[2] Sanders PTJ, Bus MPA, Scheper H, van der Wal RJP, van de Sande MAJ (2019). Multiflora and gram-negative microorganisms predominate in infections affecting pelvic endoprostheses following tumor resection. J Bone Joint Surg Am.

[3] Zmistowski B, Fedorka CJ, Sheehan E, Deirmengian G, Austin MS (2011). Prosthetic joint infection caused by gram-negative organisms. J Arthroplasty.

[4] Crémet L, Broquet A, Brulin B, Jacqueline C, Dauvergne S (2015). Pathogenic potential of *Escherichia coli* clinical strains from orthopedic implant infections towards human osteoblastic cells. Pathog Dis.

[5] Crémet L, Corvec S, Bémer P, Bret L, Lebrun C (2012). Orthopaedic-implant infections by *Escherichia coli*: molecular and phenotypic analysis of the causative strains. J Infect.

[6] Peterson J (1996). Microbiology.

[7] Idowu OJ, Onipede AO, Orimolade AE, Akinyoola LA, Babalola GO (2011). Extended-spectrum beta-lactamase orthopedic wound infections in Nigeria. J Glob Infect Dis.

[8] Picozzi SCM, Casellato S, Rossini M, Paola G, Tejada M (2014). Extended-spectrum beta-lactamase-positive *Escherichia coli* causing complicated upper urinary tract infection: urologist should act in time. Urol Ann.

[9] Müller D, Kaiser D, Sairanen K, Studhalter T, Uçkay İ (2019). Antimicrobial prophylaxis for the prevention of surgical site Infections in orthopaedic oncology - a narrative review of current concepts. J Bone Jt Infect.

[10] Ventola CL (2015). The antibiotic resistance crisis: part 1: causes and threats. P T.

[11] Tang KWK, Millar BC, Moore JE (2023). Antimicrobial resistance (AMR). Br J Biomed Sci.

[12] Schmid M, Steiner O, Fasshold L, Goessler W, Holl A-M (2020). The stability of carbapenems before and after admixture to PMMA-cement used for replacement surgery caused by gram-negative bacteria. Eur J Med Res.

[13] Kosugi K, Zenke Y, Sato N, Hamada D, Ando K (2022). Potential of continuous local antibiotic perfusion therapy for fracture-related infections. Infect Dis Ther.

[14] Shekhar C (2019). An innovative technique in local antibiotic delivery method in open infected wounds of the musculoskeletal system. Int J Low Extrem Wounds.

[15] Ter Boo GJA, Grijpma DW, Moriarty TF, Richards RG, Eglin D (2015). Antimicrobial delivery systems for local infection prophylaxis in orthopedic- and trauma surgery. Biomaterials.

[16] Wang Y, Wang Y, Wang Y, Murray CK, Hamblin MR (2017). Antimicrobial blue light inactivation of pathogenic microbes: State of the art. Drug Resist Updat.

[17] Leanse LG, Dos Anjos C, Mushtaq S, Dai T (2022). Antimicrobial blue light: a ‘magic bullet’ for the 21st century and beyond?. Adv Drug Deliv Rev.

[18] Wang Y, Ferrer-Espada R, Gu Y, Dai T (2017). Antimicrobial blue light: an alternative therapeutic for multidrug-resistant gonococcal infections?. MOJ Sol Photoenergy Syst.

[19] Wang Y, Ferrer-Espada R, Baglo Y, Goh XS, Held KD (2019). Photoinactivation of neisseria gonorrhoeae: a paradigm-changing approach for combating antibiotic-resistant gonococcal infection. J Infect Dis.

[20] Meurer L, Payne W, Guffey JS (2020). Visible light as an inhibitor of *Campylobacter jejuni*. Int J Antimicrob Agents.

[21] Mohamad SA, Milward MR, Kuehne SA, Hadis MA, Palin WM (2021). Potential for direct application of blue light for photo-disinfection of dentine. J Photochem Photobiol B.

[22] Felix Gomez GG, Lippert F, Ando M, Zandona AF, Eckert GJ (2019). Photoinhibition of *Streptococcus mutans* biofilm-induced lesions in human dentin by violet-blue light. Dent J.

[23] Tsutsumi-Arai C, Arai Y, Terada-Ito C, Imamura T, Tatehara S (2022). Microbicidal effect of 405-nm blue LED light on *Candida albicans* and *Streptococcus mutans* dual-species biofilms on denture base resin. Lasers Med Sci.

[24] Bumah VV, Masson-Meyers DS, Tong W, Castel C, Enwemeka CS (2020). Optimizing the bactericidal effect of pulsed blue light on propionibacterium acnes - a correlative fluorescence spectroscopy study. J Photochem Photobiol.

[25] Bumah VV, Masson-Meyers DS, Enwemeka CS (2020). Pulsed 450 nm blue light suppresses MRSA and propionibacterium acnes in planktonic cultures and bacterial biofilms. J Photochem Photobiol B.

[26] Brownson JRS (2014). Solar Energy Conversion Systems, First Edition.

[27] Lahuerta Zamora L, Pérez-Gracia MT (2012). Using digital photography to implement the McFarland method. J R Soc Interface.

[28] Balouiri M, Sadiki M, Ibnsouda SK (2016). Methods for *in vitro* evaluating antimicrobial activity: a review. J Pharm Anal.

[29] Leber AL (2016). Clinical Microbiology Procedures Handbook.

[30] Zadrazilova I, Pospisilova S, Pauk K, Imramovsky A, Vinsova J (2015). *In vitro* bactericidal activity of 4- and 5-chloro-2-hydroxy-N-[1-oxo-1-(phenylamino)alkan-2-yl]benzamides against MRSA. Biomed Res Int.

[31] Del Bene VE, Walker HK, Hall WD, Hurst JW (1990). Clinical Methods: The History, Physical, and Laboratory Examinations.

[32] Abbott S, Chase L, Villines M (2011). Efficacy of structural pasteurization for reduction of viable bacterial levels in indoor environments. Indoor Air.

[33] Rapacka-Zdonczyk A, Wozniak A, Kruszewska B, Waleron K, Grinholc M (2021). Can gram-negative bacteria develop resistance to antimicrobial blue light treatment?. Int J Mol Sci.

[34] Dos Anjos C, Leanse LG, Ribeiro MS, Sellera FP, Dropa M (2023). New insights into the bacterial targets of antimicrobial blue light. Microbiol Spectr.

[35] Gardner A, Soni A, Cookson A, Brightwell G (2023). Light tolerance of extended spectrum β-lactamase producing *Escherichia coli* strains after repetitive exposure to far-UVC and blue LED light. J Appl Microbiol.

[36] Dos Anjos C, Sabino CP, Bueris V, Fernandes MR, Pogliani FC (2019). Antimicrobial blue light inactivation of international clones of multidrug-resistant *Escherichia coli* ST10, ST131 and ST648. Photodiagnosis Photodyn Ther.

[37] Maclean M, MacGregor SJ, Anderson JG, Woolsey G (2009). Inactivation of bacterial pathogens following exposure to light from a 405-nanometer light-emitting diode array. Appl Environ Microbiol.

[38] Rhodes NLR, de la Presa M, Barneck MD, Poursaid A, Firpo MA (2016). Violet 405 nm light: a novel therapeutic agent against β-lactam-resistant *Escherichia coli*. Lasers Surg Med.

[39] Dos Anjos C, Wang Y, Truong-Bolduc QC, Bolduc PK, Liu M (2024). Blue light compromises bacterial β-lactamases activity to overcome β-lactam resistance. Lasers Surg Med.

[40] Dos Anjos C, Sellera FP, Ribeiro MS, Baptista MS, Pogliani FC (2020). Antimicrobial blue light and photodynamic therapy inhibit clinically relevant β-lactamases with extended-spectrum (ESBL) and carbapenemase activity. Photodiagnosis Photodyn Ther.

