# Supplementary material for: RETRACTED: Antimicrobial effect of blue light on antibiotic-sensitive and drug-resistant Escherichia coli: a novel isotropic optical fibre
Source: Access Microbiol. 2025 Mar 19;7(3):000967.v3. doi: 10.1099/acmi.0.000967.v3 (PMC11923094; doi:10.1099/acmi.0.000967.v3)
Supplement: Uncited Supplementary Material 1. [file acmi-7-00967-s001.pdf]

Blue light study: E. coli ATCC 25922 / single / double fiber; 0-6h ; 22.11.2023

E.coli 22.11.23<br>0 Min 1:10 1)<br>n.a.	E.coli 22.11.23<br>L1+2 3 h / ud 1)<br>0	E.coli 22.11.23<br>L1+2 6 h / ud 1)<br>0	E.coli 22.11.23<br>L3 1h / ud 1)<br>0	E.coli 22.11.23<br>L3 4 h/ ud 1)<br>0	E.coli 22.11.23<br>UT, 1 h / 1:10 1)<br>n.a.	E.coli 22.11.23<br>UT, 5 h / 1:10 1)<br>n.a
E.coli 22.11.23<br>0 Min 1:10 2)<br>n.a.	E.coli 22.11.23<br>L1+2 3 h / ud 2)<br>0	E.coli 22.11.23<br>L1+2 6 h / ud 2)<br>0	E.coli 22.11.23<br>L3 1h / ud 2)<br>0	E.coli 22.11.23<br>L3 4 h / ud 2)<br>0	E.coli 22.11.23<br>UT, 1 h / 1:10 2)<br>n.a.	E.coli 22.11.23<br>UT, 5 h / 1:10 2)<br>n.a
E.coli 22.11.23<br>0 Min 1:10 3)<br>n.a.	E.coli 22.11.23<br>L1+2 3 h / ud 3)<br>0	E.coli 22.11.23<br>L1+2 6 h / ud 3)<br>0	E.coli 22.11.23<br>L3 1h / ud 3)<br>0	E.coli 22.11.23<br>L3 4 h / ud 3)<br>0	E.coli 22.11.23<br>UT, 1 h / 1:10 3)<br>n.a	E.coli 22.11.23<br>UT, 5 h / 1:10 3)<br>n.a
E.coli 22.11.23<br>0 Min 1:100 1)<br>73	E.coli 22.11.23<br>L1+2 3 h / 1:10 1)<br>0	E.coli 22.11.23<br>L1+2 6 h / 1:10 1)<br>0	E.coli 22.11.23<br>L3 1h / 1:10 1)<br>0	E.coli 22.11.23<br>L3 4 h / 1:10 1)<br>0	E.coli 22.11.23<br>UT, 1 h / 1:100 1)<br>52	E.coli 22.11.23<br>UT, 5 h / 1:100 1)<br>54
E.coli 22.11.23<br>0 Min 1:100 2)<br>58	E.coli 22.11.23<br>L1+2 3 h / 1:10 2)<br>0	E.coli 22.11.23<br>L1+2 6 h / 1:10 2)<br>0	E.coli 22.11.23<br>L3 1 h / 1:10 2)<br>0	E.coli 22.11.23<br>L3 4 h / 1:10 2)<br>0	E.coli 22.11.23<br>UT, 1 h / 1:100 2)<br>50	E.coli 22.11.23<br>UT, 5 h / 1:100 2)<br>51
E.coli 22.11.23<br>0 Min 1:100 3)<br>46	E.coli 22.11.23<br>L1+2 3 h / 1:10 3)<br>0	E.coli 22.11.23<br>L1+2 6 h / 1:10 3)<br>0	E.coli 22.11.23<br>L3 1 h / 1:10 3)<br>0	E.coli 22.11.23<br>L3 4 h / 1:10 3)<br>0	E.coli 22.11.23<br>UT, 1 h / 1:100 3)<br>57	E.coli 22.11.23<br>UT, 5 h / 1:100 3)<br>56
E.coli 22.11.23<br>L1+2 1 h / ud 1)<br>0	E.coli 22.11.23<br>L1+2 3 h / 1:100 1)<br>0	E.coli 22.11.23<br>L1+2 6 h / 1:100 1)<br>0	E.coli 22.11.23<br>L3 1 h / 1:100 1)<br>0	E.coli 22.11.23<br>L3 4 h / 1:100 1)<br>0	E.coli 22.11.23<br>UT, 2 h / 1:10 1)<br>n.a.	E.coli 22.11.23<br>UT, 6 h / 1:10 1)<br>n.a
E.coli 22.11.23<br>L1+2 1 h / ud 2)<br>0	E.coli 22.11.23<br>L1+2 3 h / 1:100 2)<br>0	E.coli 22.11.23<br>L1+2 6 h / 1:100 2)<br>0	E.coli 22.11.23<br>L3 1 h / 1:100 2)<br>0	E.coli 22.11.23<br>L3 4 h / 1:100 2)<br>0	E.coli 22.11.23<br>UT, 2 h / 1:10 2)<br>n.a	E.coli 22.11.23<br>UT, 6 h / 1:10 2)<br>n.a
E.coli 22.11.23<br>L1+2 1 h / ud 3)<br>0	E.coli 22.11.23<br>L1+2 3 h / 1:100 3)<br>0	E.coli 22.11.23<br>L1+2 6 h / 1:100 3)<br>0	E.coli 22.11.23<br>L3 1 h / 1:100 3)<br>0	E.coli 22.11.23<br>L3 4 h / 1:100 3)<br>0	E.coli 22.11.23<br>UT, 2 h / 1:10 3)<br>n.a	E.coli 22.11.23<br>UT, 6 h / 1:10 3)<br>n.a
E.coli 22.11.23<br>L1+2 1 h / 1:10 1)<br>0	E.coli 22.11.23<br>L1+2 4 h / ud 1)<br>0		E.coli 22.11.23<br>L3 2 h / ud 1)<br>0	E.coli 22.11.23<br>L3 5 h / ud 1)<br>0	E.coli 22.11.23<br>UT, 2 h / 1:100 1)<br>51	E.coli 22.11.23<br>UT, 6 h / 1:100 1)<br>47
E.coli 22.11.23<br>L1+2 1 h / 1:10 2)<br>0	E.coli 22.11.23<br>L1+2 4 h / ud 2)<br>0		E.coli 22.11.23<br>L3 2 h / ud 2)<br>0	E.coli 22.11.23<br>L3 5 h / ud 2)<br>0	E.coli 22.11.23<br>UT, 2 h / 1:100 2)<br>45	E.coli 22.11.23<br>UT, 6 h / 1:100 2)<br>55
E.coli 22.11.23<br>L1+2 1 h / 1:10 3)<br>0	E.coli 22.11.23<br>L1+2 4 h / ud 3)<br>0		E.coli 22.11.23<br>L3 2 h / ud 3)<br>0	E.coli 22.11.23<br>L3 5 h / ud 3)<br>0	E.coli 22.11.23<br>UT, 2 h / 1:100 3)<br>48	E.coli 22.11.23<br>UT, 6 h / 1:100 3)<br>50
E.coli 22.11.23<br>L1+2 1 h / 1:100 1)<br>0	E.coli 22.11.23<br>L1+2 4 h / 1:10 1)<br>0		E.coli 22.11.23<br>L3 2 h / 1:10 1)<br>0	E.coli 22.11.23<br>L3 5 h / 1:10 1)<br>0	E.coli 22.11.23<br>UT, 3 h / 1:10 1)<br>n.a.	
E.coli 22.11.23<br>L1+2 1 h / 1:100 2)<br>0	E.coli 22.11.23<br>L1+2 4 h / 1:10 2)<br>0		E.coli 22.11.23<br>L3 2 h / 1:10 2)<br>0	E.coli 22.11.23<br>L3 5 h / 1:10 2)<br>0	E.coli 22.11.23<br>UT, 3 h / 1:10 2)<br>n.a.	
E.coli 22.11.23<br>L1+2 1 h / 1:100 3)<br>0	E.coli 22.11.23<br>L1+2 4 h / 1:10 3)<br>0		E.coli 22.11.23<br>L3 2 h / 1:10 3)<br>0	E.coli 22.11.23<br>L3 5 h / 1:10 3)<br>0	E.coli 22.11.23<br>UT, 3 h / 1:10 3)<br>n.a	
E.coli 22.11.23<br>L1+2 2 h / ud 1)<br>0	E.coli 22.11.23<br>L1+2 4 h / 1:100 1)<br>0		E.coli 22.11.23<br>L3 2 h / 1:100 1)<br>0	E.coli 22.11.23<br>L3 5 h / 1:100 1)<br>0	E.coli 22.11.23<br>UT, 3 h / 1:100 1)<br>48	
E.coli 22.11.23<br>L1+2 2 h / ud 2)<br>0	E.coli 22.11.23<br>L1+2 4 h / 1:100 2)<br>0		E.coli 22.11.23<br>L3 2 h / 1:100 2)<br>0	E.coli 22.11.23<br>L3 5 h / 1:100 2)<br>0	E.coli 22.11.23<br>UT, 3 h / 1:100 2)<br>42	
E.coli 22.11.23<br>L1+2 2h / ud 3)<br>0	E.coli 22.11.23<br>L1+2 4 h / 1:100 3)<br>0		E.coli 22.11.23<br>L3 2 h / 1:100 3)<br>0	E.coli 22.11.23<br>L3 5 h / 1:100 3)<br>0	E.coli 22.11.23<br>UT, 3 h / 1:100 3)<br>57	
E.coli 22.11.23<br>L1+2 2h / 1:10 1)<br>0	E.coli 22.11.23<br>L1+2 5 h / ud 1)<br>0		E.coli 22.11.23<br>L3 3 h / ud 1)<br>0	E.coli 22.11.23<br>L3 6 h / ud 1)<br>0	E.coli 22.11.23<br>UT, 4 h / 1:10 1)<br>n.a	
E.coli 22.11.23<br>L1+2 2h / 1:10 2)<br>0	E.coli 22.11.23<br>L1+2 5 h / ud 2)<br>0		E.coli 22.11.23<br>L3 3 h / ud 2)<br>0	E.coli 22.11.23<br>L3 6 h / ud 2)<br>0	E.coli 22.11.23<br>UT, 4 h / 1:10 2)<br>n.a	
E.coli 22.11.23<br>L1+2 2h / 1:10 3)<br>0	E.coli 22.11.23<br>L1+2 5 h / ud 3)<br>0		E.coli 22.11.23<br>L3 3 h / ud 3)<br>0	E.coli 22.11.23<br>L3 6 h / ud 3)<br>0	E.coli 22.11.23<br>UT, 4 h / 1:10 3)<br>n.a	
E.coli 22.11.23<br>L1+2 2h / 1:100 1)<br>0	E.coli 22.11.23<br>L1+2 5 h / 1:10 1)<br>0		E.coli 22.11.23<br>L3 3 h / 1:10 1)<br>0	E.coli 22.11.23<br>L3 6 h / 1:10 1)<br>0	E.coli 22.11.23<br>UT, 4 h / 1:100 1)<br>59	
E.coli 22.11.23<br>L1+2 2h / 1:100 2)<br>0	E.coli 22.11.23<br>L1+2 5 h / 1:10 2)<br>0		E.coli 22.11.23<br>L3 3 h / 1:10 2)<br>0	E.coli 22.11.23<br>L3 6 h / 1:10 2)<br>0	E.coli 22.11.23<br>UT, 4 h / 1:100 2)<br>43	
E.coli 22.11.23<br>L1+2 2h / 1:100 3)<br>0	E.coli 22.11.23<br>L1+2 5 h / 1:10 3)<br>0		E.coli 22.11.23<br>L3 3 h / 1:10 3)<br>0	E.coli 22.11.23<br>L3 6 h / 1:10 3)<br>0	E.coli 22.11.23<br>UT, 4 h / 1:100 3)<br>51	
	E.coli 22.11.23<br>L1+2 5 h / 1:100 1)<br>0		E.coli 22.11.23<br>L3 3 h / 1:100 1)<br>0	E.coli 22.11.23<br>L3 6 h / 1:100 1)<br>0		
	E.coli 22.11.23<br>L1+2 5 h / 1:100 2)<br>0		E.coli 22.11.23<br>L3 3 h / 1:100 2)<br>0	E.coli 22.11.23<br>L3 6 h / 1:100 2)<br>0		
	E.coli 22.11.23<br>L1+2 5 h / 1:100 3)<br>0		E.coli 22.11.23<br>L3 3 h / 1:100 3)<br>0	E.coli 22.11.23<br>L3 6 h / 1:100 3)<br>0		

Blue light study: E. coli ATCC 25922; single/double fiber; 0-6 h; 23.11.2023

E.coli 23.11.23<br>0 Min 1:10 1)<br>n.c.	E.coli 23.11.23<br>L5+6 3 h / ud 1)<br>0	E.coli 23.11.23<br>L5+6 6 h / ud 1)<br>0	E.coli 23.11.23<br>L4 1h / ud 1)<br>0	E.coli 23.11.23<br>L4 4 h/ ud 1)<br>0	E.coli 23.11.23<br>UT, 1 h / 1:10 1)<br>n.c.	E.coli 23.11.23<br>UT, 5 h / 1:10 1)<br>n.c.
E.coli 23.11.23<br>0 Min 1:10 2)<br>n.c.	E.coli 23.11.23<br>L5+6 3 h / ud 2)<br>0	E.coli 23.11.23<br>L5+6 6 h / ud 2)<br>0	E.coli 23.11.23<br>L4 1h / ud 2)<br>0	E.coli 23.11.23<br>L4 4 h / ud 2)<br>0	E.coli 23.11.23<br>UT, 1 h / 1:10 2)<br>n.c.	E.coli 23.11.23<br>UT, 5 h / 1:10 2)<br>n.c.
E.coli 23.11.23<br>0 Min 1:10 3)<br>n.c.	E.coli 23.11.23<br>L5+6 3 h / ud 3)<br>0	E.coli 23.11.23<br>L5+6 6 h / ud 3)<br>0	E.coli 23.11.23<br>L4 1h / ud 3)<br>0	E.coli 23.11.23<br>L4 4 h / ud 3)<br>0	E.coli 23.11.23<br>UT, 1 h / 1:10 3)<br>n.c.	E.coli 23.11.23<br>UT, 5 h / 1:10 3)<br>n.c.
E.coli 23.11.23<br>0 Min 1:100 1)<br>76	E.coli 23.11.23<br>L5+6 3 h / 1:10 1)<br>0	E.coli 23.11.23<br>L5+6 6 h / 1:10 1)<br>0	E.coli 23.11.23<br>L4 1h / 1:10 1)<br>0	E.coli 23.11.23<br>L4 4 h / 1:10 1)<br>0	E.coli 23.11.23<br>UT, 1 h / 1:100 1)<br>51	E.coli 23.11.23<br>UT, 5 h / 1:100 1)<br>65
E.coli 23.11.23<br>0 Min 1:100 2)<br>53	E.coli 23.11.23<br>L5+6 3 h / 1:10 2)<br>0	E.coli 23.11.23<br>L5+6 6 h / 1:10 2)<br>0	E.coli 23.11.23<br>L4 1 h / 1:10 2)<br>0	E.coli 23.11.23<br>L4 4 h / 1:10 2)<br>0	E.coli 23.11.23<br>UT, 1 h / 1:100 2)<br>54	E.coli 23.11.23<br>UT, 5 h / 1:100 2)<br>55
E.coli 23.11.23<br>0 Min 1:100 3)<br>62	E.coli 23.11.23<br>L5+6 3 h / 1:10 3)<br>0	E.coli 23.11.23<br>L5+6 6 h / 1:10 3)<br>0	E.coli 23.11.23<br>L4 1 h / 1:10 3)<br>0	E.coli 23.11.23<br>L4 4 h / 1:10 3)<br>0	E.coli 23.11.23<br>UT, 1 h / 1:100 3)<br>55	E.coli 23.11.23<br>UT, 5 h / 1:100 3)<br>59
E.coli 23.11.23<br>L5+6 1 h / ud 1)<br>0	E.coli 23.11.23<br>L5+6 3 h / 1:100 1)<br>0	E.coli 23.11.23<br>L5+6 6 h / 1:100 1)<br>0	E.coli 23.11.23<br>L4 1 h / 1:100 1)<br>0	E.coli 23.11.23<br>L4 4 h / 1:100 1)<br>0	E.coli 23.11.23<br>UT, 2 h / 1:10 1)<br>n.c.	E.coli 23.11.23<br>UT, 6 h / 1:10 1)<br>n.c.
E.coli 23.11.23<br>L5+6 1 h / ud 2)<br>0	E.coli 23.11.23<br>L5+6 3 h / 1:100 2)<br>0	E.coli 23.11.23<br>L5+6 6 h / 1:100 2)<br>0	E.coli 23.11.23<br>L4 1 h / 1:100 2)<br>0	E.coli 23.11.23<br>L4 4 h / 1:100 2)<br>0	E.coli 23.11.23<br>UT, 2 h / 1:10 2)<br>n.c.	E.coli 23.11.23<br>UT, 6 h / 1:10 2)<br>n.c.
E.coli 23.11.23<br>L5+6 1 h / ud 3)<br>0	E.coli 23.11.23<br>L5+6 3 h / 1:100 3)<br>0	E.coli 23.11.23<br>L5+6 6 h / 1:100 3)<br>0	E.coli 23.11.23<br>L4 1 h / 1:100 3)<br>0	E.coli 23.11.23<br>L4 4 h / 1:100 3)<br>0	E.coli 23.11.23<br>UT, 2 h / 1:10 3)<br>n.c.	E.coli 23.11.23<br>UT, 6 h / 1:10 3)<br>n.c.
E.coli 23.11.23<br>L5+6 1 h / 1:10 1)<br>0	E.coli 23.11.23<br>L5+6 4 h / ud 1)<br>0		E.coli 23.11.23<br>L4 2 h / ud 1)<br>0	E.coli 23.11.23<br>L4 5 h / ud 1)<br>0	E.coli 23.11.23<br>UT, 2 h / 1:100 1)<br>54	E.coli 23.11.23<br>UT, 6 h / 1:100 1)<br>57
E.coli 23.11.23<br>L5+6 1 h / 1:10 2)<br>0	E.coli 23.11.23<br>L5+6 4 h / ud 2)<br>0		E.coli 23.11.23<br>L4 2 h / ud 2)<br>0	E.coli 23.11.23<br>L4 5 h / ud 2)<br>0	E.coli 23.11.23<br>UT, 2 h / 1:100 2)<br>61	E.coli 23.11.23<br>UT, 6 h / 1:100 2)<br>62
E.coli 23.11.23<br>L5+6 1 h / 1:10 3)<br>0	E.coli 23.11.23<br>L5+6 4 h / ud 3)<br>0		E.coli 23.11.23<br>L4 2 h / ud 3)<br>0	E.coli 23.11.23<br>L4 5 h / ud 3)<br>0	E.coli 23.11.23<br>UT, 2 h / 1:100 3)<br>67	E.coli 23.11.23<br>UT, 6 h / 1:100 3)<br>56
E.coli 23.11.23<br>L5+6 1 h / 1:100 1)<br>0	E.coli 23.11.23<br>L5+6 4 h / 1:10 1)<br>0		E.coli 23.11.23<br>L4 2 h / 1:10 1)<br>0	E.coli 23.11.23<br>L4 5 h / 1:10 1)<br>0	E.coli 23.11.23<br>UT, 3 h / 1:10 1)<br>n.c.	
E.coli 23.11.23<br>L5+6 1 h / 1:100 2)<br>0	E.coli 23.11.23<br>L5+6 4 h / 1:10 2)<br>0		E.coli 23.11.23<br>L4 2 h / 1:10 2)<br>0	E.coli 23.11.23<br>L4 5 h / 1:10 2)<br>0	E.coli 23.11.23<br>UT, 3 h / 1:10 2)<br>n.c.	
E.coli 23.11.23<br>L5+6 1 h / 1:100 3)<br>0	E.coli 23.11.23<br>L5+6 4 h / 1:10 3)<br>0		E.coli 23.11.23<br>L4 2 h / 1:10 3)<br>0	E.coli 23.11.23<br>L4 5 h / 1:10 3)<br>0	E.coli 23.11.23<br>UT, 3 h / 1:10 3)<br>n.c.	
E.coli 23.11.23<br>L5+6 2 h / ud 1)<br>0	E.coli 23.11.23<br>L5+6 4 h / 1:100 1)<br>0		E.coli 23.11.23<br>L4 2 h / 1:100 1)<br>0	E.coli 23.11.23<br>L4 5 h / 1:100 1)<br>0	E.coli 23.11.23<br>UT, 3 h / 1:100 1)<br>59	
E.coli 23.11.23<br>L5+6 2 h / ud 2)<br>0	E.coli 23.11.23<br>L5+6 4 h / 1:100 2)<br>0		E.coli 23.11.23<br>L4 2 h / 1:100 2)<br>0	E.coli 23.11.23<br>L4 5 h / 1:100 2)<br>0	E.coli 23.11.23<br>UT, 3 h / 1:100 2)<br>57	
E.coli 23.11.23<br>L5+6 2h / ud 3)<br>0	E.coli 23.11.23<br>L5+6 4 h / 1:100 3)<br>0		E.coli 23.11.23<br>L4 2 h / 1:100 3)<br>0	E.coli 23.11.23<br>L4 5 h / 1:100 3)<br>0	E.coli 23.11.23<br>UT, 3 h / 1:100 3)<br>57	
E.coli 23.11.23<br>L5+6 2h / 1:10 1)<br>0	E.coli 23.11.23<br>L5+6 5 h / ud 1)<br>0		E.coli 23.11.23<br>L4 3 h / ud 1)<br>0	E.coli 23.11.23<br>L4 6 h / ud 1)<br>0	E.coli 23.11.23<br>UT, 4 h / 1:10 1)<br>n.c.	
E.coli 23.11.23<br>L5+6 2h / 1:10 2)<br>0	E.coli 23.11.23<br>L5+6 5 h / ud 2)<br>0		E.coli 23.11.23<br>L4 3 h / ud 2)<br>0	E.coli 23.11.23<br>L4 6 h / ud 2)<br>0	E.coli 23.11.23<br>UT, 4 h / 1:10 2)<br>n.c.	
E.coli 23.11.23<br>L5+6 2h / 1:10 3)<br>0	E.coli 23.11.23<br>L5+6 5 h / ud 3)<br>0		E.coli 23.11.23<br>L4 3 h / ud 3)<br>0	E.coli 23.11.23<br>L4 6 h / ud 3)<br>0	E.coli 23.11.23<br>UT, 4 h / 1:10 3)<br>n.c.	
E.coli 23.11.23<br>L5+6 2h / 1:100 1)<br>0	E.coli 23.11.23<br>L5+6 5 h / 1:10 1)<br>0		E.coli 23.11.23<br>L4 3 h / 1:10 1)<br>0	E.coli 23.11.23<br>L4 6 h / 1:10 1)<br>0	E.coli 23.11.23<br>UT, 4 h / 1:100 1)<br>57	
E.coli 23.11.23<br>L5+6 2h / 1:100 2)<br>0	E.coli 23.11.23<br>L5+6 5 h / 1:10 2)<br>0		E.coli 23.11.23<br>L4 3 h / 1:10 2)<br>0	E.coli 23.11.23<br>L4 6 h / 1:10 2)<br>0	E.coli 23.11.23<br>UT, 4 h / 1:100 2)<br>63	
E.coli 23.11.23<br>L5+6 2h / 1:100 3)<br>0	E.coli 23.11.23<br>L5+6 5 h / 1:10 3)<br>0		E.coli 23.11.23<br>L4 3 h / 1:10 3)<br>0	E.coli 23.11.23<br>L4 6 h / 1:10 3)<br>0	E.coli 23.11.23<br>UT, 4 h / 1:100 3)<br>65	
	E.coli 23.11.23<br>L5+6 5 h / 1:100 1)<br>0		E.coli 23.11.23<br>L4 3 h / 1:100 1)<br>0	E.coli 23.11.23<br>L4 6 h / 1:100 1)<br>0		
	E.coli 23.11.23<br>L5+6 5 h / 1:100 2)<br>0		E.coli 23.11.23<br>L4 3 h / 1:100 2)<br>0	E.coli 23.11.23<br>L4 6 h / 1:100 2)<br>0		
	E.coli 23.11.23<br>L5+6 5 h / 1:100 3)<br>0		E.coli 23.11.23<br>L4 3 h / 1:100 3)<br>0	E.coli 23.11.23<br>L4 6 h / 1:100 3)<br>0		

Blue light study: E. coli ATCC 25922; single / double fiber; 0 - 6 h; 29.11.2023

E.coli 29.11.23<br>0 Min 1:10 1)<br>n.p.	E.coli 29.11.23<br>L1+2 3 h / ud 1)<br>0	E.coli 29.11.23<br>L1+2 6 h / ud 1)<br>0	E.coli 29.11.23<br>L3 1h / ud 1)<br>0	E.coli 29.11.23<br>L3 4 h/ ud 1)<br>0	E.coli 29.11.23<br>UT, 1 h / 1:10 1)<br>n.p.	E.coli 29.11.23<br>UT, 5 h / 1:10 1)<br>n.p.
E.coli 29.11.23<br>0 Min 1:10 2)<br>n.p.	E.coli 29.11.23<br>L1+2 3 h / ud 2)<br>0	E.coli 29.11.23<br>L1+2 6 h / ud 2)<br>0	E.coli 29.11.23<br>L3 1h / ud 2)<br>0	E.coli 29.11.23<br>L3 4 h / ud 2)<br>0	E.coli 29.11.23<br>UT, 1 h / 1:10 2)<br>n.p.	E.coli 29.11.23<br>UT, 5 h / 1:10 2)<br>n.p.
E.coli 29.11.23<br>0 Min 1:10 3)<br>n.p.	E.coli 29.11.23<br>L1+2 3 h / ud 3)<br>0	E.coli 29.11.23<br>L1+2 6 h / ud 3)<br>0	E.coli 29.11.23<br>L3 1h / ud 3)<br>0	E.coli 29.11.23<br>L3 4 h / ud 3)<br>0	E.coli 29.11.23<br>UT, 1 h / 1:10 3)<br>n.p.	E.coli 29.11.23<br>UT, 5 h / 1:10 3)<br>n.p.
E.coli 29.11.23<br>0 Min 1:100 1)<br>50	E.coli 29.11.23<br>L1+2 3 h / 1:10 1)<br>n.p.	E.coli 29.11.23<br>L1+2 6 h / 1:10 1)<br>n.p.	E.coli 29.11.23<br>L3 1h / 1:10 1)<br>0	E.coli 29.11.23<br>L3 4 h / 1:10 1)<br>n.p.	E.coli 29.11.23<br>UT, 1 h / 1:100 1)<br>53	E.coli 29.11.23<br>UT, 5 h / 1:100 1)<br>54
E.coli 29.11.23<br>0 Min 1:100 2)<br>53	E.coli 29.11.23<br>L1+2 3 h / 1:10 2)<br>n.p.	E.coli 29.11.23<br>L1+2 6 h / 1:10 2)<br>n.p.	E.coli 29.11.23<br>L3 1 h / 1:10 2)<br>0	E.coli 29.11.23<br>L3 4 h / 1:10 2)<br>n.p.	E.coli 29.11.23<br>UT, 1 h / 1:100 2)<br>49	E.coli 29.11.23<br>UT, 5 h / 1:100 2)<br>46
E.coli 29.11.23<br>0 Min 1:100 3)<br>51	E.coli 29.11.23<br>L1+2 3 h / 1:10 3)<br>n.p.	E.coli 29.11.23<br>L1+2 6 h / 1:10 3)<br>n.p.	E.coli 29.11.23<br>L3 1 h / 1:10 3)<br>0	E.coli 29.11.23<br>L3 4 h / 1:10 3)<br>n.p.	E.coli 29.11.23<br>UT, 1 h / 1:100 3)<br>51	E.coli 29.11.23<br>UT, 5 h / 1:100 3)<br>47
E.coli 29.11.23<br>L1+2 1 h / ud 1)<br>0	E.coli 29.11.23<br>L1+2 3 h / 1:100 1)<br>n.p.	E.coli 29.11.23<br>L1+2 6 h / 1:100 1)<br>n.p.	E.coli 29.11.23<br>L3 1 h / 1:100 1)<br>n.p.	E.coli 29.11.23<br>L3 4 h / 1:100 1)<br>n.p.	E.coli 29.11.23<br>UT, 2 h / 1:10 1)<br>n.p.	E.coli 29.11.23<br>UT, 6 h / 1:10 1)<br>n.p.
E.coli 29.11.23<br>L1+2 1 h / ud 2)<br>0	E.coli 29.11.23<br>L1+2 3 h / 1:100 2)<br>n.p.	E.coli 29.11.23<br>L1+2 6 h / 1:100 2)<br>n.p.	E.coli 29.11.23<br>L3 1 h / 1:100 2)<br>n.p.	E.coli 29.11.23<br>L3 4 h / 1:100 2)<br>n.p.	E.coli 29.11.23<br>UT, 2 h / 1:10 2)<br>n.p.	E.coli 29.11.23<br>UT, 6 h / 1:10 2)<br>n.p.
E.coli 29.11.23<br>L1+2 1 h / ud 3)<br>0	E.coli 29.11.23<br>L1+2 3 h / 1:100 3)<br>n.p.	E.coli 29.11.23<br>L1+2 6 h / 1:100 3)<br>n.p.	E.coli 29.11.23<br>L3 1 h / 1:100 3)<br>n.p.	E.coli 29.11.23<br>L3 4 h / 1:100 3)<br>n.p.	E.coli 29.11.23<br>UT, 2 h / 1:10 3)<br>n.p.	E.coli 29.11.23<br>UT, 6 h / 1:10 3)<br>n.p.
E.coli 29.11.23<br>L1+2 1 h / 1:10 1)<br>0	E.coli 29.11.23<br>L1+2 4 h / ud 1)<br>0		E.coli 29.11.23<br>L3 2 h / ud 1)<br>0	E.coli 29.11.23<br>L3 5 h / ud 1)<br>0	E.coli 29.11.23<br>UT, 2 h / 1:100 1)<br>54	E.coli 29.11.23<br>UT, 6 h / 1:100 1)<br>46
E.coli 29.11.23<br>L1+2 1 h / 1:10 2)<br>0	E.coli 29.11.23<br>L1+2 4 h / ud 2)<br>0		E.coli 29.11.23<br>L3 2 h / ud 2)<br>0	E.coli 29.11.23<br>L3 5 h / ud 2)<br>0	E.coli 29.11.23<br>UT, 2 h / 1:100 2)<br>48	E.coli 29.11.23<br>UT, 6 h / 1:100 2)<br>54
E.coli 29.11.23<br>L1+2 1 h / 1:10 3)<br>0	E.coli 29.11.23<br>L1+2 4 h / ud 3)<br>0		E.coli 29.11.23<br>L3 2 h / ud 3)<br>0	E.coli 29.11.23<br>L3 5 h / ud 3)<br>0	E.coli 29.11.23<br>UT, 2 h / 1:100 3)<br>51	E.coli 29.11.23<br>UT, 6 h / 1:100 3)<br>52
E.coli 29.11.23<br>L1+2 1 h / 1:100 1)<br>n.p.	E.coli 29.11.23<br>L1+2 4 h / 1:10 1)<br>n.p.		E.coli 29.11.23<br>L3 2 h / 1:10 1)<br>n.p.	E.coli 29.11.23<br>L3 5 h / 1:10 1)<br>n.p.	E.coli 29.11.23<br>UT, 3 h / 1:10 1)<br>n.p.	
E.coli 29.11.23<br>L1+2 1 h / 1:100 2)<br>n.p.	E.coli 29.11.23<br>L1+2 4 h / 1:10 2)<br>n.p.		E.coli 29.11.23<br>L3 2 h / 1:10 2)<br>n.p.	E.coli 29.11.23<br>L3 5 h / 1:10 2)<br>n.p.	E.coli 29.11.23<br>UT, 3 h / 1:10 2)<br>n.p.	
E.coli 29.11.23<br>L1+2 1 h / 1:100 3)<br>n.p.	E.coli 29.11.23<br>L1+2 4 h / 1:10 3)<br>n.p.		E.coli 29.11.23<br>L3 2 h / 1:10 3)<br>n.p.	E.coli 29.11.23<br>L3 5 h / 1:10 3)<br>n.p.	E.coli 29.11.23<br>UT, 3 h / 1:10 3)<br>n.p.	
E.coli 29.11.23<br>L1+2 2 h / ud 1)<br>0	E.coli 29.11.23<br>L1+2 4 h / 1:100 1)<br>n.p.		E.coli 29.11.23<br>L3 2 h / 1:100 1)<br>n.p.	E.coli 29.11.23<br>L3 5 h / 1:100 1)<br>n.p.	E.coli 29.11.23<br>UT, 3 h / 1:100 1)<br>48	
E.coli 29.11.23<br>L1+2 2 h / ud 2)<br>0	E.coli 29.11.23<br>L1+2 4 h / 1:100 2)<br>n.p.		E.coli 29.11.23<br>L3 2 h / 1:100 2)<br>n.p.	E.coli 29.11.23<br>L3 5 h / 1:100 2)<br>n.p.	E.coli 29.11.23<br>UT, 3 h / 1:100 2)<br>49	
E.coli 29.11.23<br>L1+2 2h / ud 3)<br>0	E.coli 29.11.23<br>L1+2 4 h / 1:100 3)<br>n.p.		E.coli 29.11.23<br>L3 2 h / 1:100 3)<br>n.p.	E.coli 29.11.23<br>L3 5 h / 1:100 3)<br>n.p.	E.coli 29.11.23<br>UT, 3 h / 1:100 3)<br>47	
E.coli 29.11.23<br>L1+2 2h / 1:10 1)<br>n.p.	E.coli 29.11.23<br>L1+2 5 h / ud 1)<br>0		E.coli 29.11.23<br>L3 3 h / ud 1)<br>0	E.coli 29.11.23<br>L3 6 h / ud 1)<br>0	E.coli 29.11.23<br>UT, 4 h / 1:10 1)<br>n.p.	
E.coli 29.11.23<br>L1+2 2h / 1:10 2)<br>n.p.	E.coli 29.11.23<br>L1+2 5 h / ud 2)<br>0		E.coli 29.11.23<br>L3 3 h / ud 2)<br>0	E.coli 29.11.23<br>L3 6 h / ud 2)<br>0	E.coli 29.11.23<br>UT, 4 h / 1:10 2)<br>n.p.	
E.coli 29.11.23<br>L1+2 2h / 1:10 3)<br>n.p.	E.coli 29.11.23<br>L1+2 5 h / ud 3)<br>0		E.coli 29.11.23<br>L3 3 h / ud 3)<br>0	E.coli 29.11.23<br>L3 6 h / ud 3)<br>0	E.coli 29.11.23<br>UT, 4 h / 1:10 3)<br>n.p.	
E.coli 29.11.23<br>L1+2 2h / 1:100 1)<br>n.p.	E.coli 29.11.23<br>L1+2 5 h / 1:10 1)<br>n.p.		E.coli 29.11.23<br>L3 3 h / 1:10 1)<br>n.p.	E.coli 29.11.23<br>L3 6 h / 1:10 1)<br>n.p.	E.coli 29.11.23<br>UT, 4 h / 1:100 1)<br>49	
E.coli 29.11.23<br>L1+2 2h / 1:100 2)<br>n.p.	E.coli 29.11.23<br>L1+2 5 h / 1:10 2)<br>n.p.		E.coli 29.11.23<br>L3 3 h / 1:10 2)<br>n.p.	E.coli 29.11.23<br>L3 6 h / 1:10 2)<br>n.p.	E.coli 29.11.23<br>UT, 4 h / 1:100 2)<br>50	
E.coli 29.11.23<br>L1+2 2h / 1:100 3)<br>n.p.	E.coli 29.11.23<br>L1+2 5 h / 1:10 3)<br>n.p.		E.coli 29.11.23<br>L3 3 h / 1:10 3)<br>n.p.	E.coli 29.11.23<br>L3 6 h / 1:10 3)<br>n.p.	E.coli 29.11.23<br>UT, 4 h / 1:100 3)<br>47	
	E.coli 29.11.23<br>L1+2 5 h / 1:100 1)<br>n.p.		E.coli 29.11.23<br>L3 3 h / 1:100 1)<br>n.p.	E.coli 29.11.23<br>L3 6 h / 1:100 1)<br>n.p.		
	E.coli 29.11.23<br>L1+2 5 h / 1:100 2)<br>n.p.		E.coli 29.11.23<br>L3 3 h / 1:100 2)<br>n.p.	E.coli 29.11.23<br>L3 6 h / 1:100 2)<br>n.p.		
	E.coli 29.11.23<br>L1+2 5 h / 1:100 3)<br>n.p.		E.coli 29.11.23<br>L3 3 h / 1:100 3)<br>n.p.	E.coli 29.11.23<br>L3 6 h / 1:100 3)<br>n.p.		

n.p. not plated

Blue Light study: E. coli ARM; single/double fiber; 0-6h; 22.11.2023

E.coli 22.11.23<br>0 Min 1:10 1)<br>n.a.	E.coli 22.11.23<br>L5+6 3 h / ud 1)<br>0	E.coli 22.11.23<br>L5+6 6 h / ud 1)<br>0	E.coli 22.11.23<br>L4 1h / ud 1)<br>n.a	E.coli 22.11.23<br>L4 4 h/ ud 1)<br>0	E.coli 22.11.23<br>UT, 1 h / 1:10 1)<br>n.a.	E.coli 22.11.23<br>UT, 5 h / 1:10 1)<br>n.a
E.coli 22.11.23<br>0 Min 1:10 2)<br>n.a.	E.coli 22.11.23<br>L5+6 3 h / ud 2)<br>0	E.coli 22.11.23<br>L5+6 6 h / ud 2)<br>0	E.coli 22.11.23<br>L4 1h / ud 2)<br>n.a	E.coli 22.11.23<br>L4 4 h / ud 2)<br>0	E.coli 22.11.23<br>UT, 1 h / 1:10 2)<br>n.a.	E.coli 22.11.23<br>UT, 5 h / 1:10 2)<br>n.a.
E.coli 22.11.23<br>0 Min 1:10 3)<br>n.a	E.coli 22.11.23<br>L5+6 3 h / ud 3)<br>0	E.coli 22.11.23<br>L5+6 6 h / ud 3)<br>0	E.coli 22.11.23<br>L4 1h / ud 3)<br>na	E.coli 22.11.23<br>L4 4 h / ud 3)<br>0	E.coli 22.11.23<br>UT, 1 h / 1:10 3)<br>n.a.	E.coli 22.11.23<br>UT, 5 h / 1:10 3)<br>n.a
E.coli 22.11.23<br>0 Min 1:100 1)<br>55	E.coli 22.11.23<br>L5+6 3 h / 1:10 1)<br>0	E.coli 22.11.23<br>L5+6 6 h / 1:10 1)<br>0	E.coli 22.11.23<br>L4 1 h / 1:10 1)<br>14	E.coli 22.11.23<br>L4 4 h / 1:10 1)<br>0	E.coli 22.11.23<br>UT, 1 h / 1:100 1)<br>54	E.coli 22.11.23<br>UT, 5 h / 1:100 1)<br>53
E.coli 22.11.23<br>0 Min 1:100 2)<br>59	E.coli 22.11.23<br>L5+6 3 h / 1:10 2)<br>0	E.coli 22.11.23<br>L5+6 6 h / 1:10 2)<br>0	E.coli 22.11.23<br>L4 1 h / 1:10 2)<br>13	E.coli 22.11.23<br>L4 4 h / 1:10 2)<br>0	E.coli 22.11.23<br>UT, 1 h / 1:100 2)<br>57	E.coli 22.11.23<br>UT, 5 h / 1:100 2)<br>56
E.coli 22.11.23<br>0 Min 1:100 3)<br>52	E.coli 22.11.23<br>L5+6 3 h / 1:10 3)<br>0	E.coli 22.11.23<br>L5+6 6 h / 1:10 3)<br>0	E.coli 22.11.23<br>L4 1 h / 1:10 3)<br>25	E.coli 22.11.23<br>L4 4 h / 1:10 3)<br>0	E.coli 22.11.23<br>UT, 1 h / 1:100 3)<br>55	E.coli 22.11.23<br>UT, 5 h / 1:100 3)<br>55
E.coli 22.11.23<br>L5+6 1 h / ud 1)<br>0	E.coli 22.11.23<br>L5+6 3 h / 1:100 1)<br>0	E.coli 22.11.23<br>L5+6 6 h / 1:100 1)<br>0	E.coli 22.11.23<br>L4 1 h / 1:100 1)<br>3	E.coli 22.11.23<br>L4 4 h / 1:100 1)<br>0	E.coli 22.11.23<br>UT, 2 h / 1:10 1)<br>n.a.	E.coli 22.11.23<br>UT, 6 h / 1:10 1)<br>n.a.
E.coli 22.11.23<br>L5+6 1 h / ud 2)<br>2	E.coli 22.11.23<br>L5+6 3 h / 1:100 2)<br>0	E.coli 22.11.23<br>L5+6 6 h / 1:100 2)<br>0	E.coli 22.11.23<br>L4 1 h / 1:100 2)<br>3	E.coli 22.11.23<br>L4 4 h / 1:100 2)<br>0	E.coli 22.11.23<br>UT, 2 h / 1:10 2)<br>n.a.	E.coli 22.11.23<br>UT, 6 h / 1:10 2)<br>n.a
E.coli 22.11.23<br>L5+6 1 h / ud 3)<br>2	E.coli 22.11.23<br>L5+6 3 h / 1:100 3)<br>0	E.coli 22.11.23<br>L5+6 6 h / 1:100 3)<br>0	E.coli 22.11.23<br>L4 1 h / 1:100 3)<br>2	E.coli 22.11.23<br>L4 4 h / 1:100 3)<br>0	E.coli 22.11.23<br>UT, 2 h / 1:10 3)<br>n.a.	E.coli 22.11.23<br>UT, 6 h / 1:10 3)<br>n.a
E.coli 22.11.23<br>L5+6 1 h / 1:10 1)<br>0	E.coli 22.11.23<br>L5+6 4 h / ud 1)<br>0		E.coli 22.11.23<br>L4 2 h / ud 1)<br>2	E.coli 22.11.23<br>L4 5 h / ud 1)<br>0	E.coli 22.11.23<br>UT, 2 h / 1:100 1)<br>50	E.coli 22.11.23<br>UT, 6 h / 1:100 1)<br>62
E.coli 22.11.23<br>L5+6 1 h / 1:10 2)<br>0	E.coli 22.11.23<br>L5+6 4 h / ud 2)<br>0		E.coli 22.11.23<br>L4 2 h / ud 2)<br>9	E.coli 22.11.23<br>L4 5 h / ud 2)<br>0	E.coli 22.11.23<br>UT, 2 h / 1:100 2)<br>52	E.coli 22.11.23<br>UT, 6 h / 1:100 2)<br>59
E.coli 22.11.23<br>L5+6 1 h / 1:10 3)<br>0	E.coli 22.11.23<br>L5+6 4 h / ud 3)<br>0		E.coli 22.11.23<br>L4 2 h / ud 3)<br>10	E.coli 22.11.23<br>L4 5 h / ud 3)<br>0	E.coli 22.11.23<br>UT, 2 h / 1:100 3)<br>51	E.coli 22.11.23<br>UT, 6 h / 1:100 3)<br>52
E.coli 22.11.23<br>L5+6 1 h / 1:100 1)<br>0	E.coli 22.11.23<br>L5+6 4 h / 1:10 1)<br>0		E.coli 22.11.23<br>L4 2 h / 1:10 1)<br>1	E.coli 22.11.23<br>L4 5 h / 1:10 1)<br>0	E.coli 22.11.23<br>UT, 3 h / 1:10 1)<br>n.a.	
E.coli 22.11.23<br>L5+6 1 h / 1:100 2)<br>0	E.coli 22.11.23<br>L5+6 4 h / 1:10 2)<br>0		E.coli 22.11.23<br>L4 2 h / 1:10 2)<br>0	E.coli 22.11.23<br>L4 5 h / 1:10 2)<br>0	E.coli 22.11.23<br>UT, 3 h / 1:10 2)<br>n.a.	
E.coli 22.11.23<br>L5+6 1 h / 1:100 3)<br>0	E.coli 22.11.23<br>L5+6 4 h / 1:10 3)<br>0		E.coli 22.11.23<br>L4 2 h / 1:10 3)<br>3	E.coli 22.11.23<br>L4 5 h / 1:10 3)<br>0	E.coli 22.11.23<br>UT, 3 h / 1:10 3)<br>n.a.	
E.coli 22.11.23<br>L5+6 2 h / ud 1)<br>0	E.coli 22.11.23<br>L5+6 4 h / 1:100 1)<br>0		E.coli 22.11.23<br>L4 2 h / 1:100 1)<br>0	E.coli 22.11.23<br>L4 5 h / 1:100 1)<br>0	E.coli 22.11.23<br>UT, 3 h / 1:100 1)<br>54	
E.coli 22.11.23<br>L5+6 2 h / ud 2)<br>0	E.coli 22.11.23<br>L5+6 4 h / 1:100 2)<br>0		E.coli 22.11.23<br>L4 2 h / 1:100 2)<br>0	E.coli 22.11.23<br>L4 5 h / 1:100 2)<br>0	E.coli 22.11.23<br>UT, 3 h / 1:100 2)<br>56	
E.coli 22.11.23<br>L5+6 2h / ud 3)<br>0	E.coli 22.11.23<br>L5+6 4 h / 1:100 3)<br>0		E.coli 22.11.23<br>L4 2 h / 1:100 3)<br>0	E.coli 22.11.23<br>L4 5 h / 1:100 3)<br>0	E.coli 22.11.23<br>UT, 3 h / 1:100 3)<br>44	
E.coli 22.11.23<br>L5+6 2h / 1:10 1)<br>0	E.coli 22.11.23<br>L5+6 5 h / ud 1)<br>0		E.coli 22.11.23<br>L4 3 h / ud 1)<br>0	E.coli 22.11.23<br>L4 6 h / ud 1)<br>0	E.coli 22.11.23<br>UT, 4 h / 1:10 1)<br>n.a	
E.coli 22.11.23<br>L5+6 2h / 1:10 2)<br>0	E.coli 22.11.23<br>L5+6 5 h / ud 2)<br>0		E.coli 22.11.23<br>L4 3 h / ud 2)<br>0	E.coli 22.11.23<br>L4 6 h / ud 2)<br>0	E.coli 22.11.23<br>UT, 4 h / 1:10 2)<br>n.a	
E.coli 22.11.23<br>L5+6 2h / 1:10 3)<br>0	E.coli 22.11.23<br>L5+6 5 h / ud 3)<br>0		E.coli 22.11.23<br>L4 3 h / ud 3)<br>0	E.coli 22.11.23<br>L4 6 h / ud 3)<br>0	E.coli 22.11.23<br>UT, 4 h / 1:10 3)<br>n.a	
E.coli 22.11.23<br>L5+6 2h / 1:100 1)<br>0	E.coli 22.11.23<br>L5+6 5 h / 1:10 1)<br>0		E.coli 22.11.23<br>L4 3 h / 1:10 1)<br>0	E.coli 22.11.23<br>L4 6 h / 1:10 1)<br>0	E.coli 22.11.23<br>UT, 4 h / 1:100 1)<br>49	
E.coli 22.11.23<br>L5+6 2h / 1:100 2)<br>0	E.coli 22.11.23<br>L5+6 5 h / 1:10 2)<br>0		E.coli 22.11.23<br>L4 3 h / 1:10 2)<br>0	E.coli 22.11.23<br>L4 6 h / 1:10 2)<br>0	E.coli 22.11.23<br>UT, 4 h / 1:100 2)<br>54	
E.coli 22.11.23<br>L5+6 2h / 1:100 3)<br>0	E.coli 22.11.23<br>L5+6 5 h / 1:10 3)<br>0		E.coli 22.11.23<br>L4 3 h / 1:10 3)<br>0	E.coli 22.11.23<br>L4 6 h / 1:10 3)<br>0	E.coli 22.11.23<br>UT, 4 h / 1:100 3)<br>48	
	E.coli 22.11.23<br>L5+6 5 h / 1:100 1)<br>0		E.coli 22.11.23<br>L4 3 h / 1:100 1)<br>0	E.coli 22.11.23<br>L4 6 h / 1:100 1)<br>0		
	E.coli 22.11.23<br>L5+6 5 h / 1:100 2)<br>0		E.coli 22.11.23<br>L4 3 h / 1:100 2)<br>0	E.coli 22.11.23<br>L4 6 h / 1:100 2)<br>0		
	E.coli 22.11.23<br>L5+6 5 h / 1:100 3)<br>0		E.coli 22.11.23<br>L4 3 h / 1:100 3)<br>0	E.coli 22.11.23<br>L4 6 h / 1:100 3)<br>0		

Blue light study: E. coli ΔRM; single/double fiber; 0-6h; 23.11.2023

Sample	Count
E.coli 23.11.23 0 Min 1:10 1)	n.c.
E.coli 23.11.23 0 Min 1:10 2)	n.c.
E.coli 23.11.23 0 Min 1:10 3)	n.c
E.coli 23.11.23 0 Min 1:100 1)	61
E.coli 23.11.23 0 Min 1:100 2)	65
E.coli 23.11.23 0 Min 1:100 3)	67
E.coli 23.11.23 L1+2 1 h / ud 1)	1
E.coli 23.11.23 L1+2 1 h / ud 2)	0
E.coli 23.11.23 L1+2 1 h / ud 3)	3
E.coli 23.11.23 L1+2 1 h / 1:10 1)	0
E.coli 23.11.23 L1+2 1 h / 1:10 2)	0
E.coli 23.11.23 L1+2 1 h / 1:10 3)	0
E.coli 23.11.23 L1+2 1 h / 1:100 1)	0
E.coli 23.11.23 L1+2 1 h / 1:100 2)	0
E.coli 23.11.23 L1+2 1 h / 1:100 3)	0
E.coli 23.11.23 L1+2 2 h / ud 1)	0
E.coli 23.11.23 L1+2 2 h / ud 2)	0
E.coli 23.11.23 L1+2 2h / ud 3)	0
E.coli 23.11.23 L1+2 2h / 1:10 1)	0
E.coli 23.11.23 L1+2 2h / 1:10 2)	0
E.coli 23.11.23 L1+2 2h / 1:10 3)	0
E.coli 23.11.23 L1+2 2h / 1:100 1)	0
E.coli 23.11.23 L1+2 2h / 1:100 2)	0
E.coli 23.11.23 L1+2 2h / 1:100 3)	0

Sample	Count
E.coli 23.11.23 L1+2 3 h / ud 1)	0
E.coli 23.11.23 L1+2 3 h / ud 2)	0
E.coli 23.11.23 L1+2 3 h / ud 3)	0
E.coli 23.11.23 L1+2 3 h / 1:10 1)	0
E.coli 23.11.23 L1+2 3 h / 1:10 2)	0
E.coli 23.11.23 L1+2 3 h / 1:10 3)	0
E.coli 23.11.23 L1+2 3 h / 1:100 1)	0
E.coli 23.11.23 L1+2 3 h / 1:100 2)	0
E.coli 23.11.23 L1+2 3 h / 1:100 3)	0
E.coli 23.11.23 L1+2 4 h / ud 1)	0
E.coli 23.11.23 L1+2 4 h / ud 2)	0
E.coli 23.11.23 L1+2 4 h / ud 3)	0
E.coli 23.11.23 L1+2 4 h / 1:10 1)	0
E.coli 23.11.23 L1+2 4 h / 1:10 2)	0
E.coli 23.11.23 L1+2 4 h / 1:10 3)	0
E.coli 23.11.23 L1+2 4 h / 1:100 1)	0
E.coli 23.11.23 L1+2 4 h / 1:100 2)	0
E.coli 23.11.23 L1+2 4 h / 1:100 3)	0
E.coli 23.11.23 L1+2 5 h / ud 1)	0
E.coli 23.11.23 L1+2 5 h / ud 2)	0
E.coli 23.11.23 L1+2 5 h / ud 3)	0
E.coli 23.11.23 L1+2 5 h / 1:10 1)	0
E.coli 23.11.23 L1+2 5 h / 1:10 2)	0
E.coli 23.11.23 L1+2 5 h / 1:10 3)	0
E.coli 23.11.23 L1+2 5 h / 1:100 1)	0
E.coli 23.11.23 L1+2 5 h / 1:100 2)	0
E.coli 23.11.23 L1+2 5 h / 1:100 3)	0

Sample	Count
E.coli 23.11.23 L1+2 6 h / ud 1)	0
E.coli 23.11.23 L1+2 6 h / ud 2)	0
E.coli 23.11.23 L1+2 6 h / ud 3)	0
E.coli 23.11.23 L1+2 6 h / 1:10 1)	0
E.coli 23.11.23 L1+2 6 h / 1:10 2)	0
E.coli 23.11.23 L1+2 6 h / 1:10 3)	0
E.coli 23.11.23 L1+2 6 h / 1:100 1)	0
E.coli 23.11.23 L1+2 6 h / 1:100 2)	0
E.coli 23.11.23 L1+2 6 h / 1:100 3)	0

Sample	Count
E.coli 23.11.23 L3 1h / ud 1)	n.c.
E.coli 23.11.23 L3 1h / ud 2)	n.c.
E.coli 23.11.23 L3 1h / ud 3)	n.c.
E.coli 23.11.23 L3 1h / 1:10 1)	13
E.coli 23.11.23 L3 1 h / 1:10 2)	13
E.coli 23.11.23 L3 1 h / 1:10 3)	18
E.coli 23.11.23 L3 1 h / 1:100 1)	3
E.coli 23.11.23 L3 1 h / 1:100 2)	3
E.coli 23.11.23 L3 1 h / 1:100 3)	0
E.coli 23.11.23 L3 2 h / ud 1)	2
E.coli 23.11.23 L3 2 h / ud 2)	3
E.coli 23.11.23 L3 2 h / ud 3)	2
E.coli 23.11.23 L3 2 h / 1:10 1)	1
E.coli 23.11.23 L3 2 h / 1:10 2)	0
E.coli 23.11.23 L3 2 h / 1:10 3)	0
E.coli 23.11.23 L3 2 h / 1:100 1)	0
E.coli 23.11.23 L3 2 h / 1:100 2)	0
E.coli 23.11.23 L3 2 h / 1:100 3)	0
E.coli 23.11.23 L3 3 h / ud 1)	0
E.coli 23.11.23 L3 3 h / ud 2)	0
E.coli 23.11.23 L3 3 h / ud 3)	0
E.coli 23.11.23 L3 3 h / 1:10 1)	0
E.coli 23.11.23 L3 3 h / 1:10 2)	0
E.coli 23.11.23 L3 3 h / 1:10 3)	0
E.coli 23.11.23 L3 3 h / 1:100 1)	0
E.coli 23.11.23 L3 3 h / 1:100 2)	0
E.coli 23.11.23 L3 3 h / 1:100 3)	0

Sample	Count
E.coli 23.11.23 L3 4 h/ ud 1)	0
E.coli 23.11.23 L3 4 h / ud 2)	0
E.coli 23.11.23 L3 4 h / ud 3)	0
E.coli 23.11.23 L3 4 h / 1:10 1)	0
E.coli 23.11.23 L3 4 h / 1:10 2)	0
E.coli 23.11.23 L3 4 h / 1:10 3)	0
E.coli 23.11.23 L3 4 h / 1:100 1)	0
E.coli 23.11.23 L3 4 h / 1:100 2)	0
E.coli 23.11.23 L3 4 h / 1:100 3)	0
E.coli 23.11.23 L3 5 h / ud 1)	0
E.coli 23.11.23 L3 5 h / ud 2)	0
E.coli 23.11.23 L3 5 h / ud 3)	0
E.coli 23.11.23 L3 5 h / 1:10 1)	0
E.coli 23.11.23 L3 5 h / 1:10 2)	0
E.coli 23.11.23 L3 5 h / 1:10 3)	0
E.coli 23.11.23 L3 5 h / 1:100 1)	0
E.coli 23.11.23 L3 5 h / 1:100 2)	0
E.coli 23.11.23 L3 5 h / 1:100 3)	0
E.coli 23.11.23 L3 6 h / ud 1)	0
E.coli 23.11.23 L3 6 h / ud 2)	9
E.coli 23.11.23 L3 6 h / ud 3)	0
E.coli 23.11.23 L3 6 h / 1:10 1)	0
E.coli 23.11.23 L3 6 h / 1:10 2)	0
E.coli 23.11.23 L3 6 h / 1:10 3)	0
E.coli 23.11.23 L3 6 h / 1:100 1)	0
E.coli 23.11.23 L3 6 h / 1:100 2)	0
E.coli 23.11.23 L3 6 h / 1:100 3)	0

Sample	Count
E.coli 23.11.23 UT, 1 h / 1:10 1)	n.c.
E.coli 23.11.23 UT, 1 h / 1:10 2)	n.c.
E.coli 23.11.23 UT, 1 h / 1:10 3)	n.c.
E.coli 23.11.23 UT, 1 h / 1:100 1)	52
E.coli 23.11.23 UT, 1 h / 1:100 2)	56
E.coli 23.11.23 UT, 1 h / 1:100 3)	59
E.coli 23.11.23 UT, 2 h / 1:10 1)	n.c.
E.coli 23.11.23 UT, 2 h / 1:10 2)	n.c.
E.coli 23.11.23 UT, 2 h / 1:10 3)	n.c.
E.coli 23.11.23 UT, 2 h / 1:100 1)	64
E.coli 23.11.23 UT, 2 h / 1:100 2)	60
E.coli 23.11.23 UT, 2 h / 1:100 3)	61
E.coli 23.11.23 UT, 3 h / 1:10 1)	n.c.
E.coli 23.11.23 UT, 3 h / 1:10 2)	n.c.
E.coli 23.11.23 UT, 3 h / 1:10 3)	n.c.
E.coli 23.11.23 UT, 3 h / 1:100 1)	57
E.coli 23.11.23 UT, 3 h / 1:100 2)	54
E.coli 23.11.23 UT, 3 h / 1:100 3)	55
E.coli 23.11.23 UT, 4 h / 1:10 1)	n.c.
E.coli 23.11.23 UT, 4 h / 1:10 2)	n.c.
E.coli 23.11.23 UT, 4 h / 1:10 3)	n.c.
E.coli 23.11.23 UT, 4 h / 1:100 1)	6.9
E.coli 23.11.23 UT, 4 h / 1:100 2)	53
E.coli 23.11.23 UT, 4 h / 1:100 3)	56

Sample	Count
E.coli 23.11.23 UT, 5 h / 1:10 1)	n.c.
E.coli 23.11.23 UT, 5 h / 1:10 2)	n.c.
E.coli 23.11.23 UT, 5 h / 1:10 3)	n.c.
E.coli 23.11.23 UT, 5 h / 1:100 1)	56
E.coli 23.11.23 UT, 5 h / 1:100 2)	53
E.coli 23.11.23 UT, 5 h / 1:100 3)	54
E.coli 23.11.23 UT, 6 h / 1:10 1)	n.c.
E.coli 23.11.23 UT, 6 h / 1:10 2)	n.c.
E.coli 23.11.23 UT, 6 h / 1:10 3)	n.c.
E.coli 23.11.23 UT, 6 h / 1:100 1)	50
E.coli 23.11.23 UT, 6 h / 1:100 2)	56
E.coli 23.11.23 UT, 6 h / 1:100 3)	60

Blue light study: E.coli ARM; single/double fiber; 0-6h; 29.11.2023

E.coli 29.11.23<br>0 Min 1:10 1)<br>n.p.	E.coli 29.11.23<br>L5+6 3 h / ud 1)<br>0	E.coli 29.11.23<br>L5+6 6 h / ud 1)<br>0	E.coli 29.11.23<br>L4 1h / ud 1)<br>n.c.	E.coli 29.11.23<br>L4 4 h/ ud 1)<br>0	E.coli 29.11.23<br>UT, 1 h / 1:10 1)<br>n.p.	E.coli 29.11.23<br>UT, 5 h / 1:10 1)<br>n.p.
E.coli 29.11.23<br>0 Min 1:10 2)<br>n.p.	E.coli 29.11.23<br>L5+6 3 h / ud 2)<br>0	E.coli 29.11.23<br>L5+6 6 h / ud 2)<br>0	E.coli 29.11.23<br>L4 1h / ud 2)<br>n.c.	E.coli 29.11.23<br>L4 4 h / ud 2)<br>0	E.coli 29.11.23<br>UT, 1 h / 1:10 2)<br>n.p.	E.coli 29.11.23<br>UT, 5 h / 1:10 2)<br>n.p.
E.coli 29.11.23<br>0 Min 1:10 3)<br>n.p.	E.coli 29.11.23<br>L5+6 3 h / ud 3)<br>0	E.coli 29.11.23<br>L5+6 6 h / ud 3)<br>0	E.coli 29.11.23<br>L4 1h / ud 3)<br>n.c.	E.coli 29.11.23<br>L4 4 h / ud 3)<br>0	E.coli 29.11.23<br>UT, 1 h / 1:10 3)<br>n.p.	E.coli 29.11.23<br>UT, 5 h / 1:10 3)<br>n.p.
E.coli 29.11.23<br>0 Min 1:100 1)<br>69	E.coli 29.11.23<br>L5+6 3 h / 1:10 1)<br>n.p.	E.coli 29.11.23<br>L5+6 6 h / 1:10 1)<br>n.p.	E.coli 29.11.23<br>L4 1h / 1:10 1)<br>28	E.coli 29.11.23<br>L4 4 h / 1:10 1)<br>n.p.	E.coli 29.11.23<br>UT, 1 h / 1:100 1)<br>62	E.coli 29.11.23<br>UT, 5 h / 1:100 1)<br>58
E.coli 29.11.23<br>0 Min 1:100 2)<br>55	E.coli 29.11.23<br>L5+6 3 h / 1:10 2)<br>n.p.	E.coli 29.11.23<br>L5+6 6 h / 1:10 2)<br>n.p.	E.coli 29.11.23<br>L4 1 h / 1:10 2)<br>29	E.coli 29.11.23<br>L4 4 h / 1:10 2)<br>n.p.	E.coli 29.11.23<br>UT, 1 h / 1:100 2)<br>53	E.coli 29.11.23<br>UT, 5 h / 1:100 2)<br>56
E.coli 29.11.23<br>0 Min 1:100 3)<br>70	E.coli 29.11.23<br>L5+6 3 h / 1:10 3)<br>n.p.	E.coli 29.11.23<br>L5+6 6 h / 1:10 3)<br>n.p.	E.coli 29.11.23<br>L4 1 h / 1:10 3)<br>32	E.coli 29.11.23<br>L4 4 h / 1:10 3)<br>n.p.	E.coli 29.11.23<br>UT, 1 h / 1:100 3)<br>52	E.coli 29.11.23<br>UT, 5 h / 1:100 3)<br>55
E.coli 29.11.23<br>L5+6 1 h / ud 1)<br>2	E.coli 29.11.23<br>L5+6 3 h / 1:100 1)<br>n.p.	E.coli 29.11.23<br>L5+6 6 h / 1:100 1)<br>n.p.	E.coli 29.11.23<br>L4 1 h / 1:100 1)<br>3	E.coli 29.11.23<br>L4 4 h / 1:100 1)<br>n.p.	E.coli 29.11.23<br>UT, 2 h / 1:10 1)<br>n.p.	E.coli 29.11.23<br>UT, 6 h / 1:10 1)<br>n.p.
E.coli 29.11.23<br>L5+6 1 h / ud 2)<br>1	E.coli 29.11.23<br>L5+6 3 h / 1:100 2)<br>n.p.	E.coli 29.11.23<br>L5+6 6 h / 1:100 2)<br>n.p.	E.coli 29.11.23<br>L4 1 h / 1:100 2)<br>4	E.coli 29.11.23<br>L4 4 h / 1:100 2)<br>n.p.	E.coli 29.11.23<br>UT, 2 h / 1:10 2)<br>n.p.	E.coli 29.11.23<br>UT, 6 h / 1:10 2)<br>n.p.
E.coli 29.11.23<br>L5+6 1 h / ud 3)<br>2	E.coli 29.11.23<br>L5+6 3 h / 1:100 3)<br>n.p.	E.coli 29.11.23<br>L5+6 6 h / 1:100 3)<br>n.p.	E.coli 29.11.23<br>L4 1 h / 1:100 3)<br>8	E.coli 29.11.23<br>L4 4 h / 1:100 3)<br>n.p.	E.coli 29.11.23<br>UT, 2 h / 1:10 3)<br>n.p.	E.coli 29.11.23<br>UT, 6 h / 1:10 3)<br>n.p.
E.coli 29.11.23<br>L5+6 1 h / 1:10 1)<br>0	E.coli 29.11.23<br>L5+6 4 h / ud 1)<br>0		E.coli 29.11.23<br>L4 2 h / ud 1)<br>7	E.coli 29.11.23<br>L4 5 h / ud 1)<br>0	E.coli 29.11.23<br>UT, 2 h / 1:100 1)<br>57	E.coli 29.11.23<br>UT, 6 h / 1:100 1)<br>68
E.coli 29.11.23<br>L5+6 1 h / 1:10 2)<br>0	E.coli 29.11.23<br>L5+6 4 h / ud 2)<br>0		E.coli 29.11.23<br>L4 2 h / ud 2)<br>9	E.coli 29.11.23<br>L4 5 h / ud 2)<br>0	E.coli 29.11.23<br>UT, 2 h / 1:100 2)<br>51	E.coli 29.11.23<br>UT, 6 h / 1:100 2)<br>55
E.coli 29.11.23<br>L5+6 1 h / 1:10 3)<br>1	E.coli 29.11.23<br>L5+6 4 h / ud 3)<br>0		E.coli 29.11.23<br>L4 2 h / ud 3)<br>7	E.coli 29.11.23<br>L4 5 h / ud 3)<br>0	E.coli 29.11.23<br>UT, 2 h / 1:100 3)<br>65	E.coli 29.11.23<br>UT, 6 h / 1:100 3)<br>59
E.coli 29.11.23<br>L5+6 1 h / 1:100 1)<br>n.p.	E.coli 29.11.23<br>L5+6 4 h / 1:10 1)<br>n.p.		E.coli 29.11.23<br>L4 2 h / 1:10 1)<br>1	E.coli 29.11.23<br>L4 5 h / 1:10 1)<br>n.p.	E.coli 29.11.23<br>UT, 3 h / 1:10 1)<br>n.p.	
E.coli 29.11.23<br>L5+6 1 h / 1:100 2)<br>n.p.	E.coli 29.11.23<br>L5+6 4 h / 1:10 2)<br>n.p.		E.coli 29.11.23<br>L4 2 h / 1:10 2)<br>0	E.coli 29.11.23<br>L4 5 h / 1:10 2)<br>n.p.	E.coli 29.11.23<br>UT, 3 h / 1:10 2)<br>n.p.	
E.coli 29.11.23<br>L5+6 1 h / 1:100 3)<br>n.p.	E.coli 29.11.23<br>L5+6 4 h / 1:10 3)<br>n.p.		E.coli 29.11.23<br>L4 2 h / 1:10 3)<br>2	E.coli 29.11.23<br>L4 5 h / 1:10 3)<br>n.p.	E.coli 29.11.23<br>UT, 3 h / 1:10 3)<br>n.p.	
E.coli 29.11.23<br>L5+6 2 h / ud 1)<br>0	E.coli 29.11.23<br>L5+6 4 h / 1:100 1)<br>n.p.		E.coli 29.11.23<br>L4 2 h / 1:100 1)<br>1	E.coli 29.11.23<br>L4 5 h / 1:100 1)<br>n.p.	E.coli 29.11.23<br>UT, 3 h / 1:100 1)<br>61	
E.coli 29.11.23<br>L5+6 2 h / ud 2)<br>0	E.coli 29.11.23<br>L5+6 4 h / 1:100 2)<br>n.p.		E.coli 29.11.23<br>L4 2 h / 1:100 2)<br>0	E.coli 29.11.23<br>L4 5 h / 1:100 2)<br>n.p.	E.coli 29.11.23<br>UT, 3 h / 1:100 2)<br>59	
E.coli 29.11.23<br>L5+6 2h / ud 3)<br>0	E.coli 29.11.23<br>L5+6 4 h / 1:100 3)<br>n.p.		E.coli 29.11.23<br>L4 2 h / 1:100 3)<br>0	E.coli 29.11.23<br>L4 5 h / 1:100 3)<br>n.p.	E.coli 29.11.23<br>UT, 3 h / 1:100 3)<br>70	
E.coli 29.11.23<br>L5+6 2h / 1:10 1)<br>n.p.	E.coli 29.11.23<br>L5+6 5 h / ud 1)<br>0		E.coli 29.11.23<br>L4 3 h / ud 1)<br>0	E.coli 29.11.23<br>L4 6 h / ud 1)<br>0	E.coli 29.11.23<br>UT, 4 h / 1:10 1)<br>n.p.	
E.coli 29.11.23<br>L5+6 2h / 1:10 2)<br>n.p.	E.coli 29.11.23<br>L5+6 5 h / ud 2)<br>0		E.coli 29.11.23<br>L4 3 h / ud 2)<br>0	E.coli 29.11.23<br>L4 6 h / ud 2)<br>0	E.coli 29.11.23<br>UT, 4 h / 1:10 2)<br>n.p.	
E.coli 29.11.23<br>L5+6 2h / 1:10 3)<br>n.p.	E.coli 29.11.23<br>L5+6 5 h / ud 3)<br>0		E.coli 29.11.23<br>L4 3 h / ud 3)<br>2	E.coli 29.11.23<br>L4 6 h / ud 3)<br>0	E.coli 29.11.23<br>UT, 4 h / 1:10 3)<br>n.p.	
E.coli 29.11.23<br>L5+6 2h / 1:100 1)<br>n.p.	E.coli 29.11.23<br>L5+6 5 h / 1:10 1)<br>n.p.		E.coli 29.11.23<br>L4 3 h / 1:10 1)<br>0	E.coli 29.11.23<br>L4 6 h / 1:10 1)<br>n.p.	E.coli 29.11.23<br>UT, 4 h / 1:100 1)<br>58	
E.coli 29.11.23<br>L5+6 2h / 1:100 2)<br>n.p.	E.coli 29.11.23<br>L5+6 5 h / 1:10 2)<br>n.p.		E.coli 29.11.23<br>L4 3 h / 1:10 2)<br>0	E.coli 29.11.23<br>L4 6 h / 1:10 2)<br>n.p.	E.coli 29.11.23<br>UT, 4 h / 1:100 2)<br>59	
E.coli 29.11.23<br>L5+6 2h / 1:100 3)<br>n.p.	E.coli 29.11.23<br>L5+6 5 h / 1:10 3)<br>n.p.		E.coli 29.11.23<br>L4 3 h / 1:10 3)<br>1	E.coli 29.11.23<br>L4 6 h / 1:10 3)<br>n.p.	E.coli 29.11.23<br>UT, 4 h / 1:100 3)<br>56	
	E.coli 29.11.23<br>L5+6 5 h / 1:100 1)<br>n.p.		E.coli 29.11.23<br>L4 3 h / 1:100 1)<br>n.p.	E.coli 29.11.23<br>L4 6 h / 1:100 1)<br>n.p.		
	E.coli 29.11.23<br>L5+6 5 h / 1:100 2)<br>n.p.		E.coli 29.11.23<br>L4 3 h / 1:100 2)<br>n.p.	E.coli 29.11.23<br>L4 6 h / 1:100 2)<br>n.p.		
	E.coli 29.11.23<br>L5+6 5 h / 1:100 3)<br>n.p.		E.coli 29.11.23<br>L4 3 h / 1:100 3)<br>n.p.	E.coli 29.11.23<br>L4 6 h / 1:100 3)<br>n.p.		

n.p. = not plated   n.c. = not countable
